# Supplementary material for: A cytotoxic T cell inspired oncolytic nanosystem promotes lytic cell death by lipid peroxidation and elicits antitumor immune responses
Source: Nat Commun. 2023 Sep 6;14:5456. doi: 10.1038/s41467-023-41335-1 (PMC10482857; doi:10.1038/s41467-023-41335-1)
Supplement: Supplementary file 1 — Supplementary Information [file 41467_2023_41335_MOESM1_ESM.pdf]

Supplementary Information

A Cytotoxic T Cell Inspired Oncolytic Nanosystem Promotes Lytic Cell Death by Lipid Peroxidation And Elicits Antitumor Immune Responses

Zhigui Zuo[1,*], Hao Yin[2,*], Yu Zhang[3], Congying Xie[3] and Qinyang Wang[2,3,#]

[1]Department of Colorectal Surgery, The First Affiliated Hospital of Wenzhou Medical University, Wenzhou, Zhejiang, P.R. China.

[2] Institute for Advanced Research, Wenzhou Medical University, Wenzhou, Zhejiang, P.R. China.

[3]The Second Affiliated Hospital and Yuying Childrens Hospital of Wenzhou Medical University, Wenzhou, Zhejiang, P.R. China.

* These authors contributed equally.

# Correspondence should be addressed to Q.W. (wangqy@wmu.edu.cn)

Corresponding author:

Qinyang Wang, Ph.D.
Wenzhou Medical University, Wenzhou
Higher Education Park
Zhejiang, P.R. China, 325035

Supplementary Methods

Material and Methods

Unless specified, chemicals were purchased from Sigma-Aldrich and used without further purification. Tetraethyl orthosilicate (TEOS), $YCl_3 \cdot 6H_2O$, $YbCl_3 \cdot 6H_2O$, $TmCl_3 \cdot 6H_2O$, oleic acid, 1-octadecene, triethanolamine (TEA), sodium chloride (NaCl) and (3-aminopropyl)-triethoxysilane (APTES) were purchased from Sigma-Aldrich. Chlorin e6 (Ce6) were purchased from MedChemExpress.

Data reporting

No statistical methods were used to predetermine the sample size. The experiments were not randomized, and the investigators were not blinded to the outcome assessment.

Preparation and characterization of the TIOs

Preparation of TIOs. Preparation of RSL3@azo-Ce6-mSiO_2-UCNPs. azo-Ce6-mSiO_2-UCNPs were synthesized according to a published procedure with some minor modifications. In brief, $YCl_3 \cdot 6H_2O$ (482.3 mg, 1.59 mmol), $YbCl_3 \cdot 6H_2O$ (155.0 mg, 0.4 mmol), and $TmCl_3 \cdot 6H_2O$ (2.75 mg, 0.01 mmol) in deionized water were added to a flask containing 15 ml oleic acid and 30 ml 1-octadecene. The solution was heated and stirred under an argon atmosphere to get rid of water to obtain a uniform yellow liquid. Then 10 ml methanol solution of NH_4F (296.3 mg, 8 mmol) and NaOH (200 mg, 5 mmol) was added and the solution was stirred at room temperature for 1 h. After methanol evaporated, the solution was heated to 300 °C and kept for 1.5 h before it was cooled down to room temperature. The mixtures were first precipitated by the addition of 20 ml ethanol, and collected by centrifugation at 10000 r/min for 10 min. Product was re-dispersed with 5 ml cyclohexane and precipitated by adding 15 ml ethanol, then collected by the same centrifugation. After 4 times washing, the UCNP was re-dispersed in 20 ml cyclohexane. Then, 800 μmol $YCl_3 \cdot 6H_2O$ in water solution was added into a flask containing 15 ml oleic acid and 30 ml 1-octadecene. The solution was heated and stirred under an argon atmosphere to get rid of water to obtain a uniform yellow liquid. The system was then cooled down to room temperature with the

flowing of argon. Then, 5 ml pre-prepared UCNP (dispersed in cyclohexane) was added and kept for another 30 min before heated to 80 °C to remove cyclohexane. Then, 10 ml methanol solution of NH4F (1 mmol) and NaOH (1.685 mmol) was added and the solution was stirred at room temperature for 2 h. After methanol evaporated, the solution was heated to 290 °C and kept for 1.5 h before it was cooled down to room temperature. The same washing steps were followed and sample was re-dispersed in 20 ml cyclohexane to obtain UCNPS. Ce6 (2 mg), EDC (6 mg), APTES (12 μl), and NHS (4 mg) in DMSO (0.5 ml) were added to a flask. The solution was stirred at room temperature for 2 h to obtain APTES-Ce6. Hexadecyl trimethyl ammonium chloride (CTAC, 0.2 g) and 2 ml UCNPS were dissolved in turn in 10 ml water at 80 °C under intensive stirring. After 8 h, 2 ml methanol solution of tetraethyl orthosilicate (0.5 ml) was added dropwise and the resulting mixture was stirred for another 1 h. Then, 0.5 ml APTES- Ce6 was added and the solution was stirred at room temperature for 24 h. The Ce6-mSiO_2-UCNPs were collected by centrifugation and washed for several times with ethanol to remove the residual reactants. Ce6-mSiO_2-UCNPs was suspended in a solution containing ethanol (10 ml) and N-(3-triethoxysilyl)-propyl-4-phenylazobenzamide (0.08 g), and the suspension was stirred at 80 °C for 1 h. The product was collected by centrifugation at 13000 r/min for 15 min. azo-Ce6-mSiO_2-UCNPs and RSL3 solutions (mass ratio of azo-Ce6-mSiO_2-UCNPs: RSL3 = 1:1) were stirred at room temperature for 24 h to reach the equilibrium state. The RSL3@azo-Ce6-mSiO_2-UCNPs was collected by centrifugation at 7000 rpm for 15 min and was washed three times with water to remove the physically adsorbed RSL3.

Transmission electron microscopy (TEM) images were acquired on a JEM-2100F electron microscope operating at 200 kV. Standard TEM samples were prepared by dropping dilute products onto carbon coated copper grids. The size distributions and zeta potential of nanoparticles were characterized by dynamic light scattering (DLS, Malvern Zetasizer Nano S90). Upconversion luminescence emission spectra were recorded on FluoroLog-3 Spectrofluorometer (Jobin Yvon, France), with the excitation of a 450 W xenon lamp and an external 0 ~ 1 W adjustable 980 nm semiconductor

laser (Beijing Hi-tech Optoelectronic Co., China). Characterization of Ce6 and azo linkages by ultraviolet-visible (UV-Vis, UV-3101PC Shimadzu spectroscope) spectroscopy.

Then, 300 μg ml^{-1} RSL3@azo-Ce6-$mSiO_2$-UCNPs were incubated with Raw 264.7 cells (~ 10^7 cells/dish) for 6 h, followed by gentle washing with PBS or DMEM complete medium to generate the precursor of TIOs. the precursor of TIOs (~ 10^7 cells/tube, 1.0 ml) were immersed in liquid nitrogen, and after 24 h, they were thawed at 37 °C, centrifuged at 1000 rpm for 3 min, washed and resuspended in DMEM complete medium or PBS to generate TIOs (5 x 10^6 units/tube, 1.0 ml). The procedure used to generate RhB load TIOs or BMDM version TIOs was similar to that used for TIOs.

Preparation of TIOs-Fe_3O_4

Preparation of Fe_3O_4-DHCA. Monodisperse Fe_3O_4 was prepared from ferric acetylacetone in oleic acid by high temperature thermal decomposition. Iron (III) acetylacetonate (12 mmol) and oleic acid (38 mmol) were added to benzyl ether (50 mL) and stirred magnetically under nitrogen for 30 minutes. The reaction mixture was slowly heated to 165°C for 30 min, followed by reflux at 280°C under nitrogen for another 30 min. Once the reaction is over, cool the black-brown mixture to room temperature. Clean the precipitate with ethanol 3 times and collect the precipitate under the condition of external magnet. The hydrophobic monodisperse Fe_3O_4 is transferred to the aqueous phase by ligand exchange reaction to obtain Fe_3O_4-DHCA. 50 mg DHCA was dissolved in 6 mL tetrahydrofuran (THF) in a three-necked flask (25 mL). The solution is heated to 50°C under argon. Then, 18 mg of monodisperse Fe_3O_4 was dispersed in 1 mL of THF and added drop by drop. After reaction for 3 h, it was cooled to room temperature, and Fe_3O_4-DHCA were precipitated by adding 500 μL NaOH (0.5 M). Precipitation is collected by centrifugation (5000 rpm/min) and redispersed in water.

Preparation of TIOs-Fe_3O_4. TIOs-Fe_3O_4 is prepared in a similar way to TIOs. The mixture of Fe_3O_4-DHCA (300 μg ml^{-1}) and RSL3@azo-Ce6-mSiO2-UCNPs (300 μg/ml) was incubated with Raw 264.7 cells (~ 10^7 cells/dish) for 1, 2, 3, and 4 h and then

gently washed in DMEM complete medium to produce TIOs-Fe_3O_4 precursors. The precursors of Fe_3O_4-TIOs were soaked in liquid nitrogen for 24 h, defrosted at 37 ℃, centrifuged at 1000 rpm for 3 min, washed and then resuspended in DMEM complete medium or PBS to produce TIOs-Fe_3O_4.

Cell lines and cell culture conditions

After preparing a single-cell suspension from mouse spleen, $CD8^+$ T cells were negatively selected by IPHASE Mouse (ICR/CD1) $CD8^+$ T Cells Isolation Kit (iPhase Biosciences). Isolated $CD8^+$ T cells were cultured in 96-well flat bottom plates with precoated anti-CD3 (1μg/mL; clone 145-2C11; BD Biosciences) and soluble anti-CD28 (1 μg/mL; clone 37.51; BD Biosciences) for 48 hours. $CD8^+$ T cells were added with complete RPMI1640 media with 10% FBS, 1% penicillin, 100 μg/mL streptomycin, 1 mM sodium pyruvate (Gibco), and nonessential amino acids (Gibco). $CD8^+$ T cells were used in experiments responding to review comments.

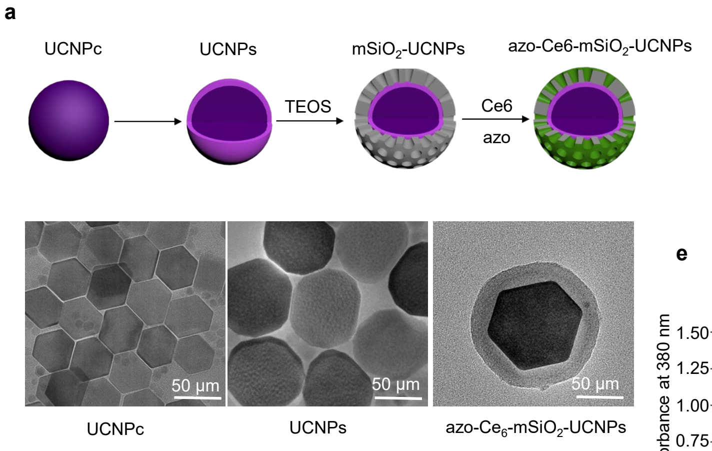

b

Sample	average size (μm)	zeta potential (mV)
UCNPc	23.70	- 10.78
UCNPs	46.4	- 28.40
azo-Ce6-mSiO$_2$-UCNPs	110.61	- 16.00

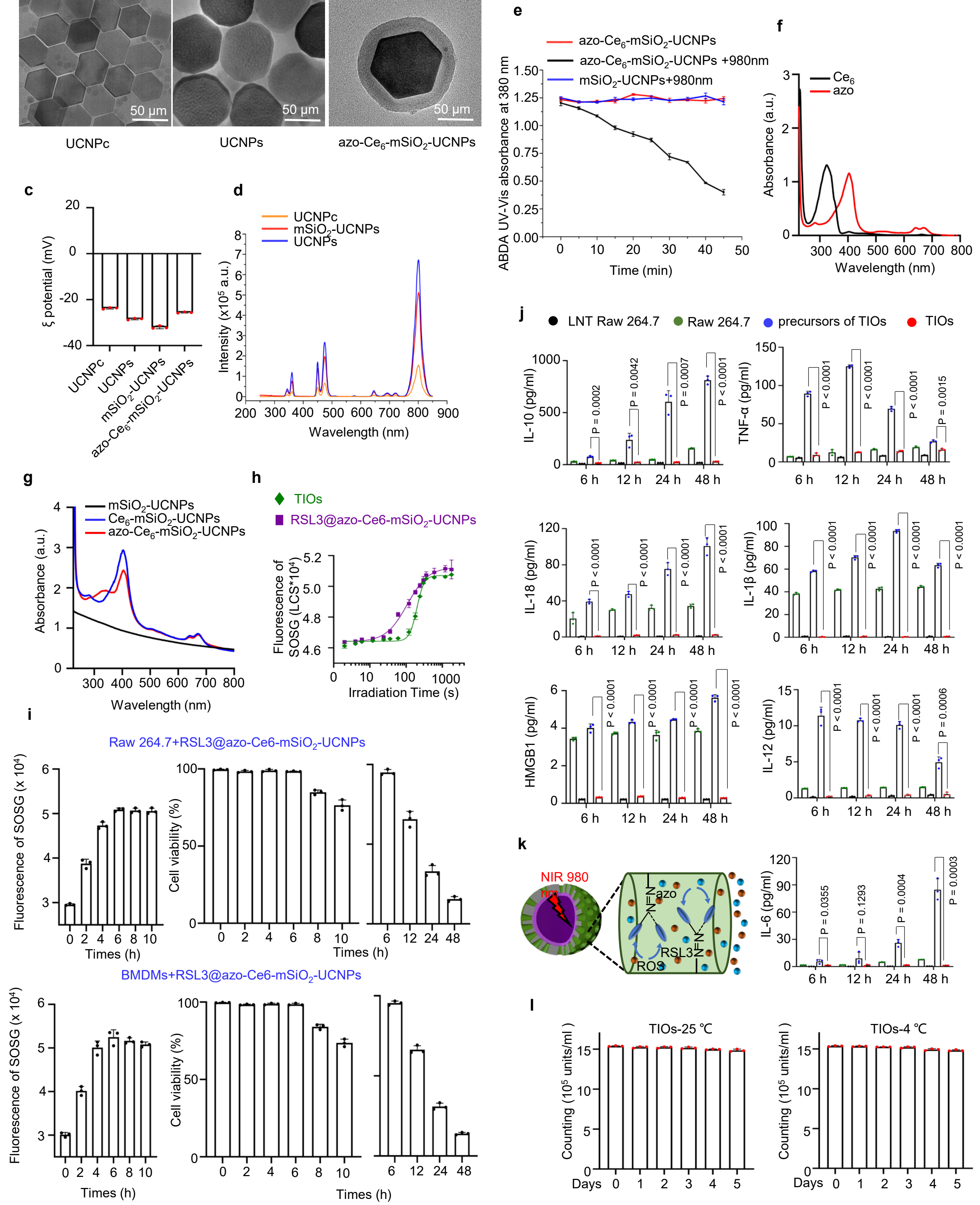

Supplementary Figure 1

Supplementary Figure 1. Synthesis and characterization of TIOs. a, The synthesis route of azo-Ce6-mSiO2-UCNPs (**top**) and Representative TEM images of UCNPc, UCNPs and azo-Ce_6-$mSiO_2$-UCNPs (**bottom**, scale bar = 50 nm, data shown are representative of two independent experiments.). **b-c,** Zeta potentials and the average size of UCNPc, UCNPs, azo-Ce_6-$mSiO_2$-UCNPs. Data are shown as mean ± s.d. (n = 3 independent experiments). **d,** Emission spectra of UCNPc, UCNPs, $mSiO_2$-UCNPs from UV to NIR light at the same concentration upon diode laser light exposure at 980 nm. **e,** Singlet oxygen generation by $mSiO_2$-UCNPs with NIR, Ce6-$mSiO_2$-UCNPs with and without NIR laser irradiation determined using an ABDA probe. Data are shown as mean ± s.d. (n = 3 independent experiments). **f,** UV/Vis absorption spectra of Ce_6 and azo. **g**, UV/Vis absorption spectra of $mSiO_2$-UCNPs, Ce_6-$mSiO_2$-UCNPs, azo-Ce_6-$mSiO_2$-UCNPs. **h,** The ROS was detected by SOSG probe with continues NIR light. **i,** RSL3@azo-Ce6-$mSiO_2$-UCNPs (300 μg ml^{-1}) was mixed in fresh DMEM complete medium and then added to Raw 264.7 (**top**) or iBMDM (**bottom**) ($1x10^7$ cells/dish) and incubated at 37 ° C for indicated time points (2 h, 4 h, 6 h, 8 h, 10 h). After washing with PBS, Singlet oxygen generation was determined using the Singlet Oxygen Sensor Green reagent to show the loading. Meanwhile, cell viability was tested with CCK8 at indicated time points (2 h, 4 h, 6 h, 8 h, 10 h, 12 h, 24 h and 48 h). Data shown as mean ± s.d. (n = 3 independent experiments, two-tailed unpaired Student's t-test). **j,** Secretion of TNF-α, IL-1β and HMGB1 from Raw 264.7, LNT Raw 264.7 (liquid nitrogen treated: LNT), the precursors of TIOs or TIOs ($1x10^6$ units/dish) at different times (6 h, 12 h, 24 h, and 48 h) were tested by corresponding ELISA kits. Data shown as mean ± s.d. (n = 3 independent experiments, two-tailed unpaired Student's t-test). **k**, Schematic diagram of ROS and RSL3 released by NIR light control in artificial granules. **l**, The stability of TIOs in PBS at 25° C or 4° C. The quantity variation of TIOs was determined by cell counter assay (10^5 units / tube). Data are shown as mean ± s.d. (n = 3 independent experiments, two-tailed unpaired Student's t-test). For **a (bottom), d, f, g,** experiment was repeated three times independently with similar results. Source data are provided as a Source Data file.

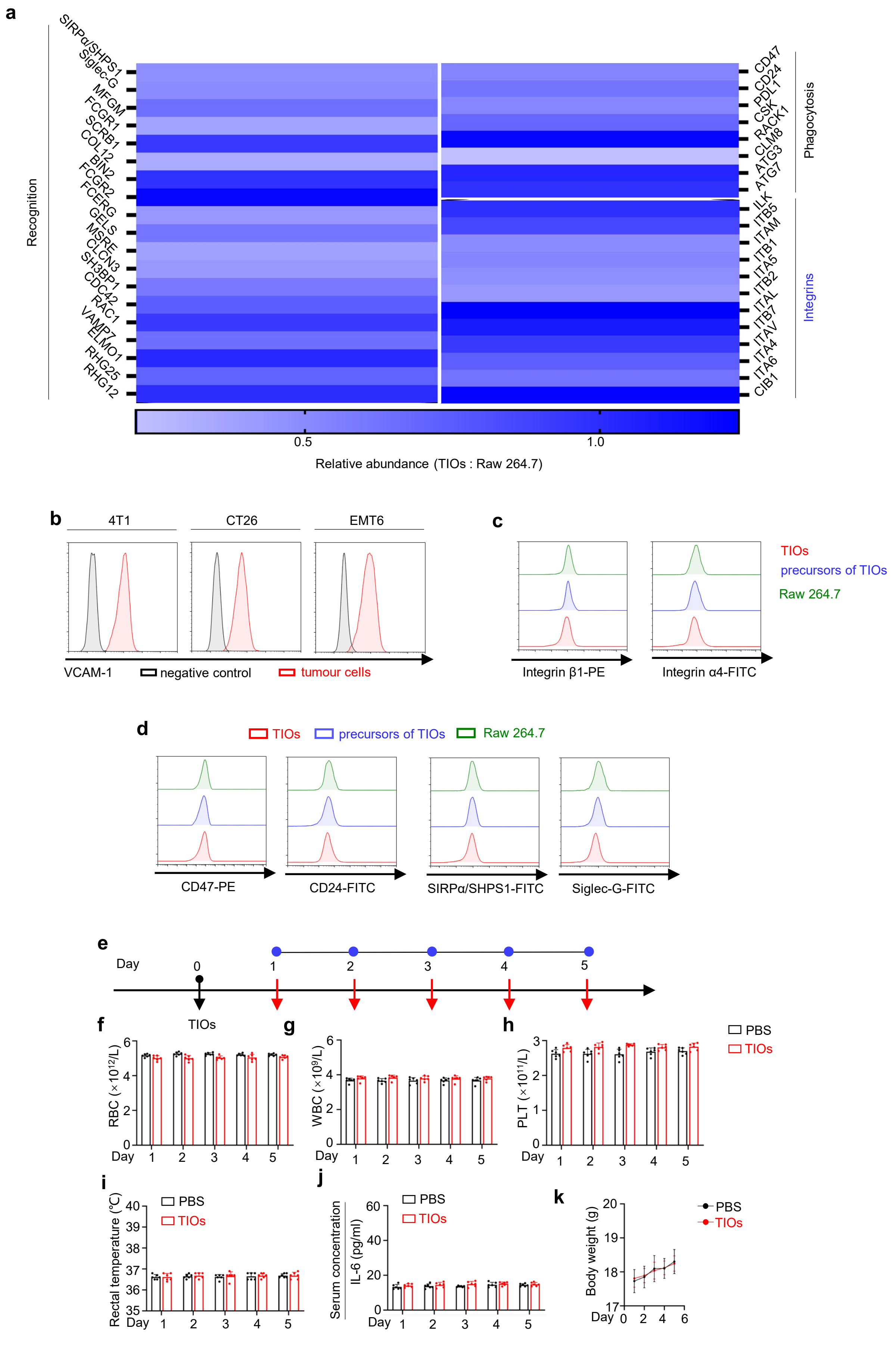

Supplementary Figure 2

Supplementary Figure 2. Characterization of binding and membrane proteins of TIOs. a, Mass spectrometric analysis of membrane proteins in Raw 264.7 cells and TIOs. **b-d**, Flow cytometry of the antigen expression. **b**, VCAM-1 on 4T1, CT26 and EMT6 cells. **c**, integrins in TIOs, precursors of TIOs and Raw 264.7. **d**, Flow cytometry of representative proteins preservation to phagocytosis in the precursors of TIOs and TIOs. **e-k**, Testing the TIOs mediated systemic inflammatory risk in mice. Analysis of systemic inflammatory response in BALB/c female mice treated with TIOs. n = 6 mice for indicated groups. **e,** TIOs mediated systemic inflammatory response scheme in BALB/c female mice. On day 1, 2, 3, 4 and 5 after intravenously injection of TIOs (5 x 10^6 units/mouse), blood was gained from the eyeballs of mice. **f-h,** The RBC (**f**), WBC (**g**) and PLT (**h**) of mice were measured at the indicated time. **i,** The rectal temperature of mice was measured at the indicated time. **j,** The serum IL-6 concentration of mice. **k,** Body weight of mice. All data in **f-k** are shown as mean ± s.e.m; two-tailed unpaired Student's t-test was performed. For **a-d**, experiment was repeated three times independently with similar results. Source data are provided as a Source Data file.

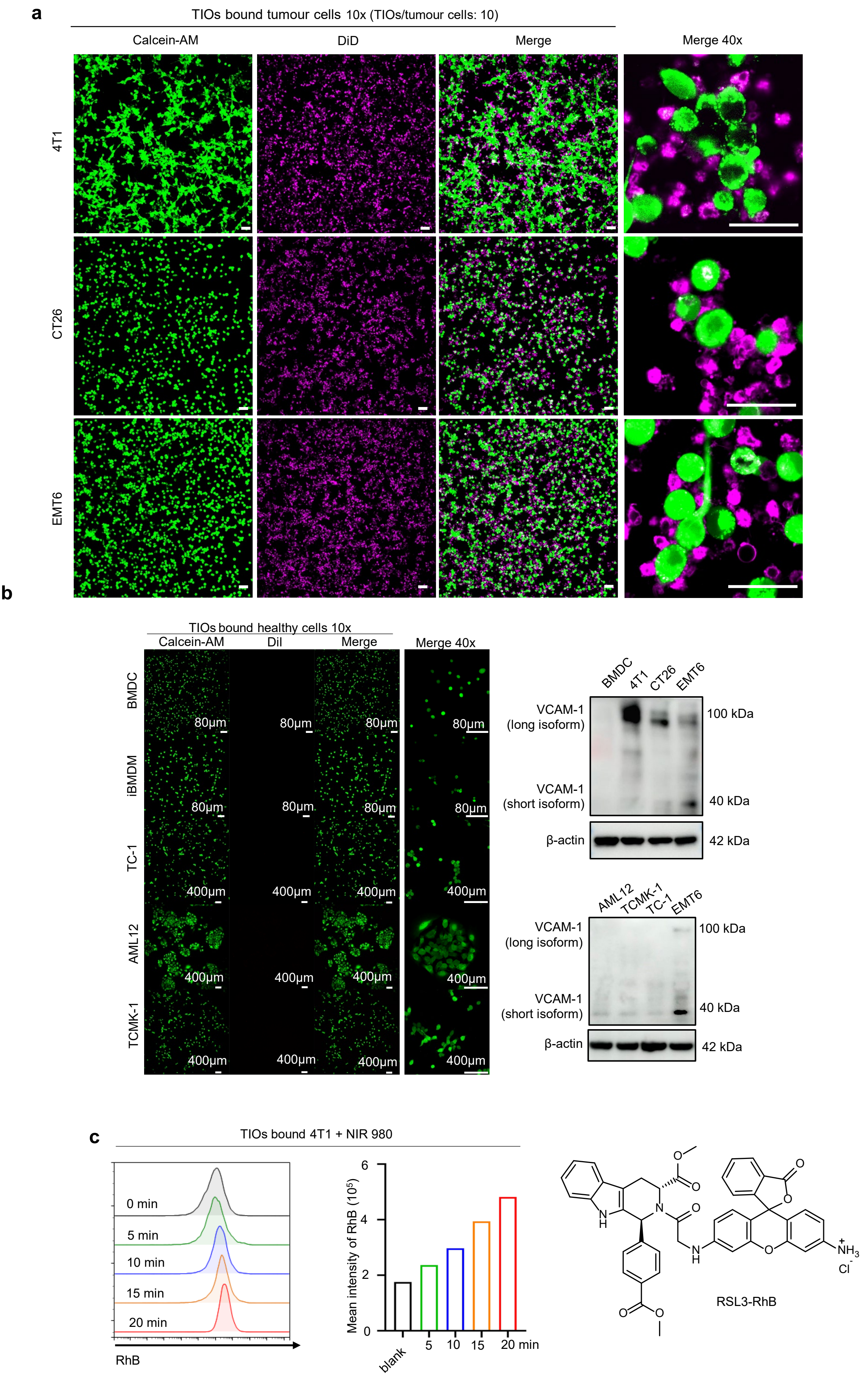

Supplementary Figure 3

Supplementary Figure 3. Binding and the payload transportation between excessive TIOs and tumour cells. a, Confocal images of the binding between DiD-labelled TIOs and Calcein-AM-labelled tumour cells. scale bar = 20 nm. TIOs : tumour cells = 10 : 1. **b**, Confocal images of catalytic amount of DiI labelled TIOs (red) treated Calcein-AM-labelled BMDCs, iBMDMs, or TC-1, AML12, TCMK-1 (green). Western blot of the VCAM-1 expression in indicated cell lines (**right**). **c**, The FACS analysis of RhB positive TIOs bound 4T1 cells. The TIOs bound 4T1 cells were irradiated by the indicated time, then the FACS was used to test the fluorescence. Data shown are representative of three independent experiments. Source data are provided as a Source Data file.

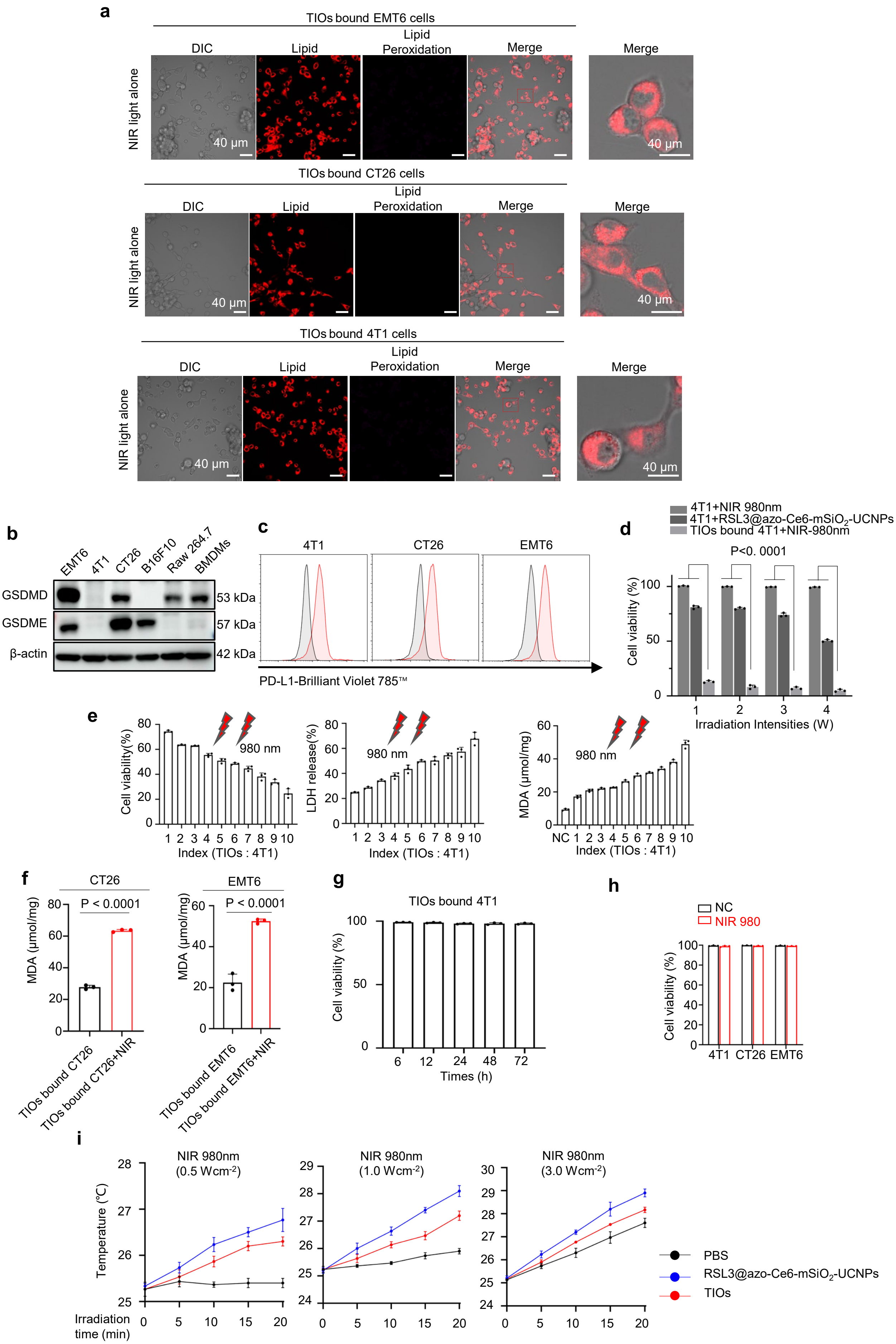

Supplementary Figure 4

Supplementary Figure 4. Cell death assays. a, Cell morphology and lipid oxidation after NIR light treatment. **b,** The western bolt of indicated cells. **c**, Flow cytometry of PD-L1 in tumour cells (4T1, CT26 and EMT6). **d**, The CCK8 assays of different NIR light intensity in indicated groups**. e,** The CCK8, LDH and MDA assays in in different index of TIOs to 4T1 cells after NIR light treatment. **f**, The MDA of TIOs bound CT26 (**left**) or TIOs bound EMT6 cells (**right**) with or without NIR light treatments. **g**, The cell viability of TIOs bound 4T1 cells for 72 hours. **h**, Cytotoxicity of NIR 980nm to tumour cells (4T1, CT26 and EMT6). Cell viability of NIR 980nm-treated tumour cells. **i**, The heating curve *in vitro.* The temperature of PBS, RSL3@azo-Ce6-mSiO2-UCNPs and TIOs treated 4T1 tumour bearing mice with the indicated NIR light. n = 3 independent experiments (**d-i**). Data (**d-i**) are shown as mean ± s.e.m; two-tailed unpaired Student's t-test was performed. Data (**a-c**) shown are representative of three independent experiments. Source data are provided as a Source Data file.

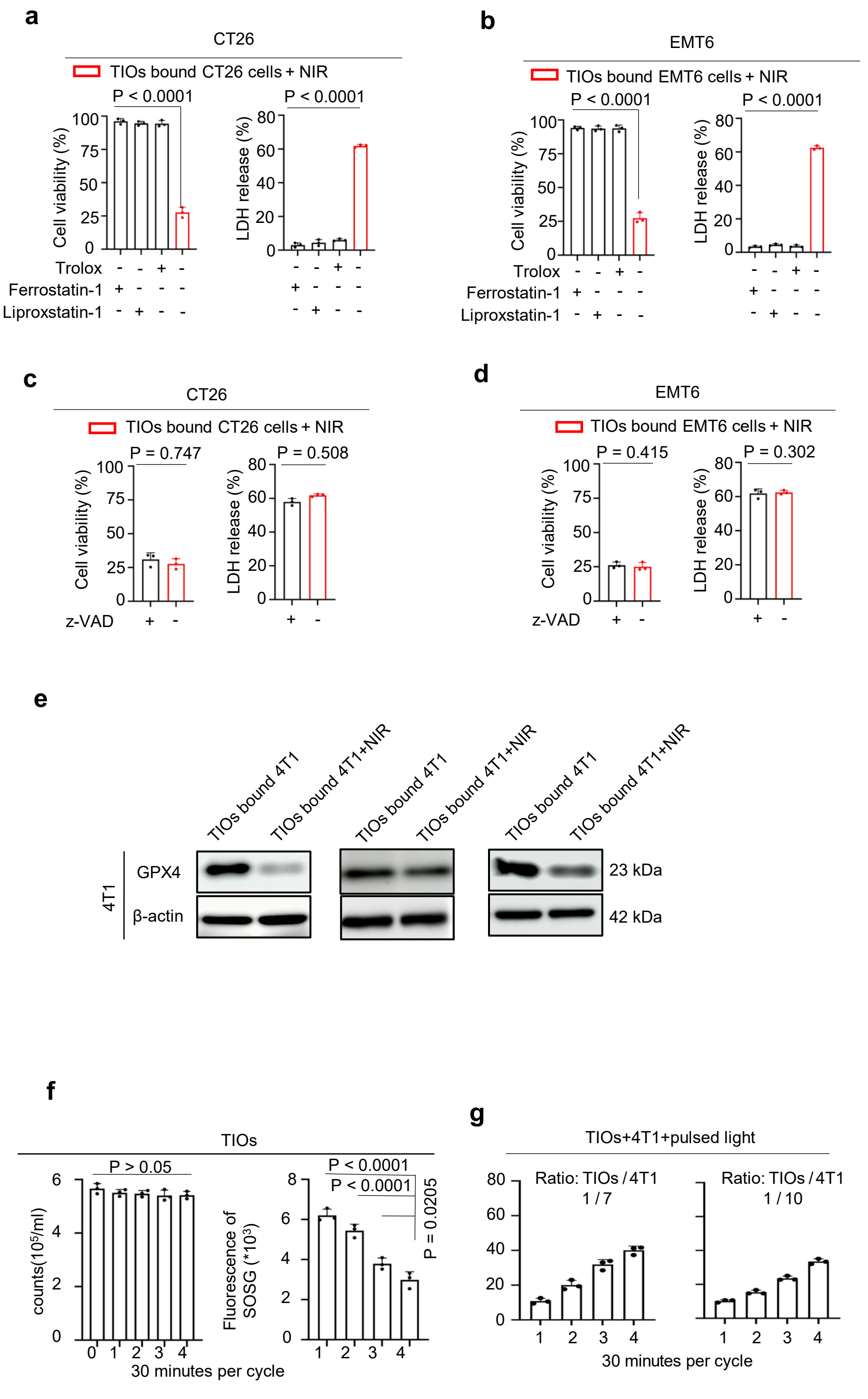

Supplementary Figure 5

Supplementary Figure 5. The mechanism of TIOs mediated cell lysis and the catalytic manner. a-d, The mechanism of TIOs mediated CT26 and EMT6 cells lysis. **a-b,** The CCK8 and LDH assays in antioxidants inhibition. **c-d,** The CCK8 and LDH assays in z-VAD inhibition. **e,** Three independent samples for testing GPX4 expression in the indicated tumour cells by immunoblotting. **f-g,** The catalytic killing ability of TIOs mediated cell lysis. **f,** Light tolerance and stability of TIOs. **g,** The LDH release of catalytic amount of TIOs mediated cell lysis with pulsed NIR light input. Data (**a-d, f-g**) are shown as mean ± s.e.m (n = 3 independent experiments); two-tailed unpaired Student's t-test was performed. Source data are provided as a Source Data file.

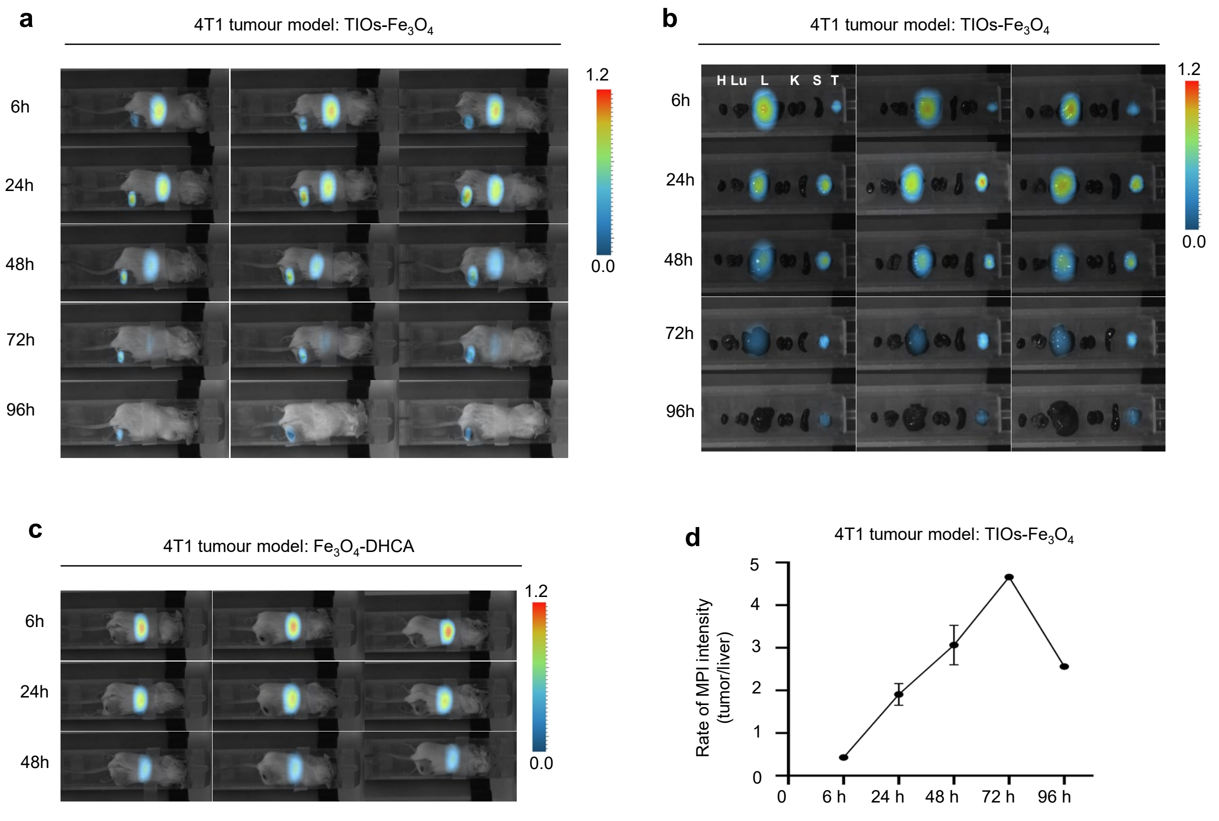

Supplementary Figure 6

Supplementary Figure 6. Biodistribution of TIOs in 4T1 mice. a The MPI images of three independent 4T1 tumour bearing mice intravenously injected with TIOs-Fe_3O_4 for 96 hours. **b** Average intensity of TIOs-Fe_3O_4 in the corresponding organs of the indicated tumour models at a series of time points. **c,** The MPI images of three independent 4T1 tumour bearing mice intravenously injected with Fe_3O_4-DHCA for 48 hours. **d,** The tumour to liver ratio of MPI intensity at different time points. Data are shown as mean ± s.d. (n = 3 mice; two-tailed unpaired Student's t-test was performed). Data shown are representative of two independent experiments. Source data are provided as a Source Data file.

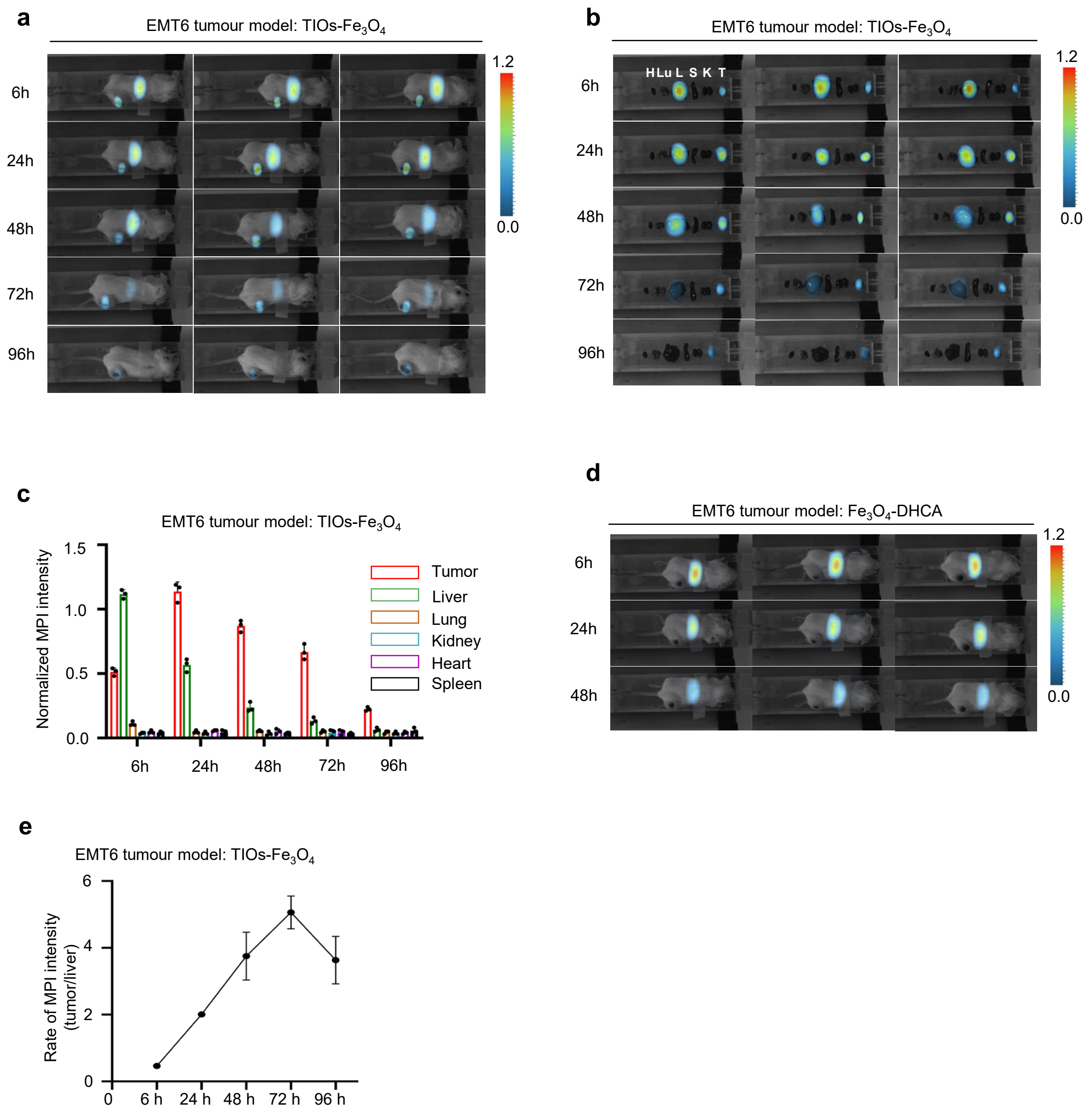

Supplementary Figure 7

Supplementary Figure 7. Biodistribution of TIOs in EMT6 mice. a, The MPI images of three independent EMT6 tumour bearing mice intravenously injected with TIOs-Fe_3O_4 for 96 hours. **b,** Average intensity of TIOs-Fe_3O_4 in the corresponding organs of the indicated tumour models at a series of time points. **c**, The biodistribution of TIOs at different time points in EMT6 tumour bearing mice. H, heart, Lu, lung, S, spleen, L, liver, K, kidney, T, tumour. (n = 3 mice). **d,** The MPI images of three independent EMT6 tumour bearing mice intravenously injected with Fe_3O_4-DHCA for 48 hours. **e**, The tumour to liver ratio of MPI intensity at different time points. Data (**c, e**) are shown as mean ± s.d. (n = 3 mice; two-tailed unpaired Student's t-test was performed). Data shown are representative of two independent experiments. Source data are provided as a Source Data file.

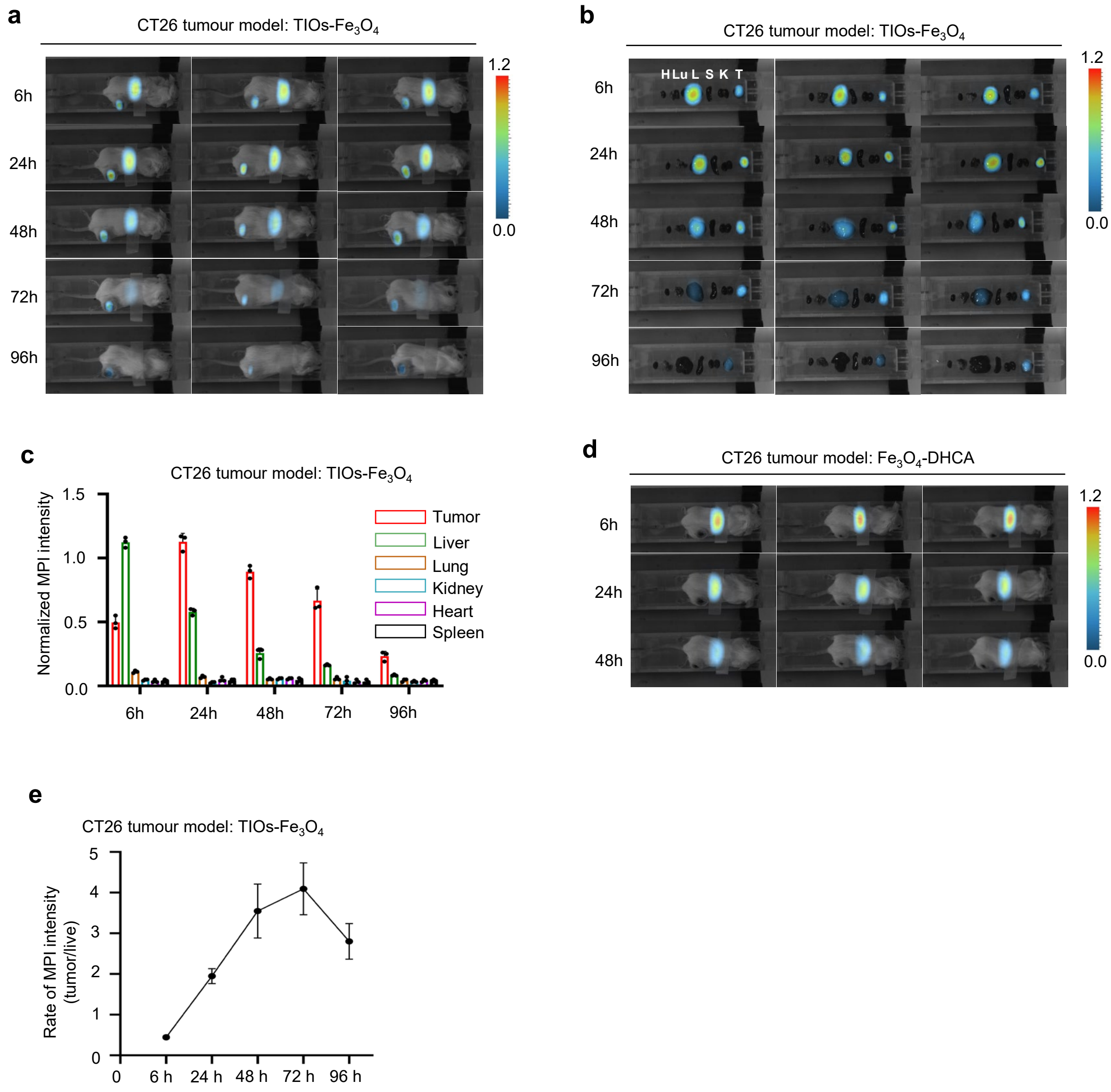

Supplementary Figure 8

Supplementary Figure 8. Biodistribution of TIOs in CT26 mice. a, The MPI images of three independent CT26 tumour bearing mice intravenously injected with TIOs-Fe_3O_4 for 96 hours. **b,** Average intensity of TIOs-Fe_3O_4 in the corresponding organs of the indicated tumour models at a series of time points. **c**, The biodistribution of TIOs at different time points in CT26 tumour bearing mice. H, heart, Lu, lung, S, spleen, L, liver, K, kidney, T, tumour. (n = 3 mice) **d,** The MPI images of three independent CT26 tumour bearing mice intravenously injected with Fe_3O_4-DHCA for 48 hours. **e**, The tumour to liver ratio of MPI intensity at different time points. Data (**c, e**) are shown as mean ± s.d. (n = 3 mice; two-tailed unpaired Student's t-test was performed). Data shown are representative of two independent experiments. Source data are provided as a Source Data file.

a

4T1 tumour model: Dil labelled RSL3@azo-Ce6-mSiO$_2$-UCNPs

Time / Ratio	12 h	24 h	48 h	72 h
Tumour / Liver	0.32	0.51	0.50	0.82

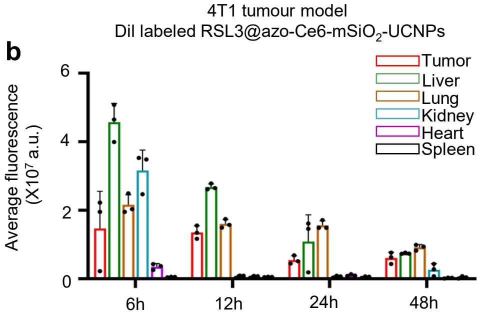

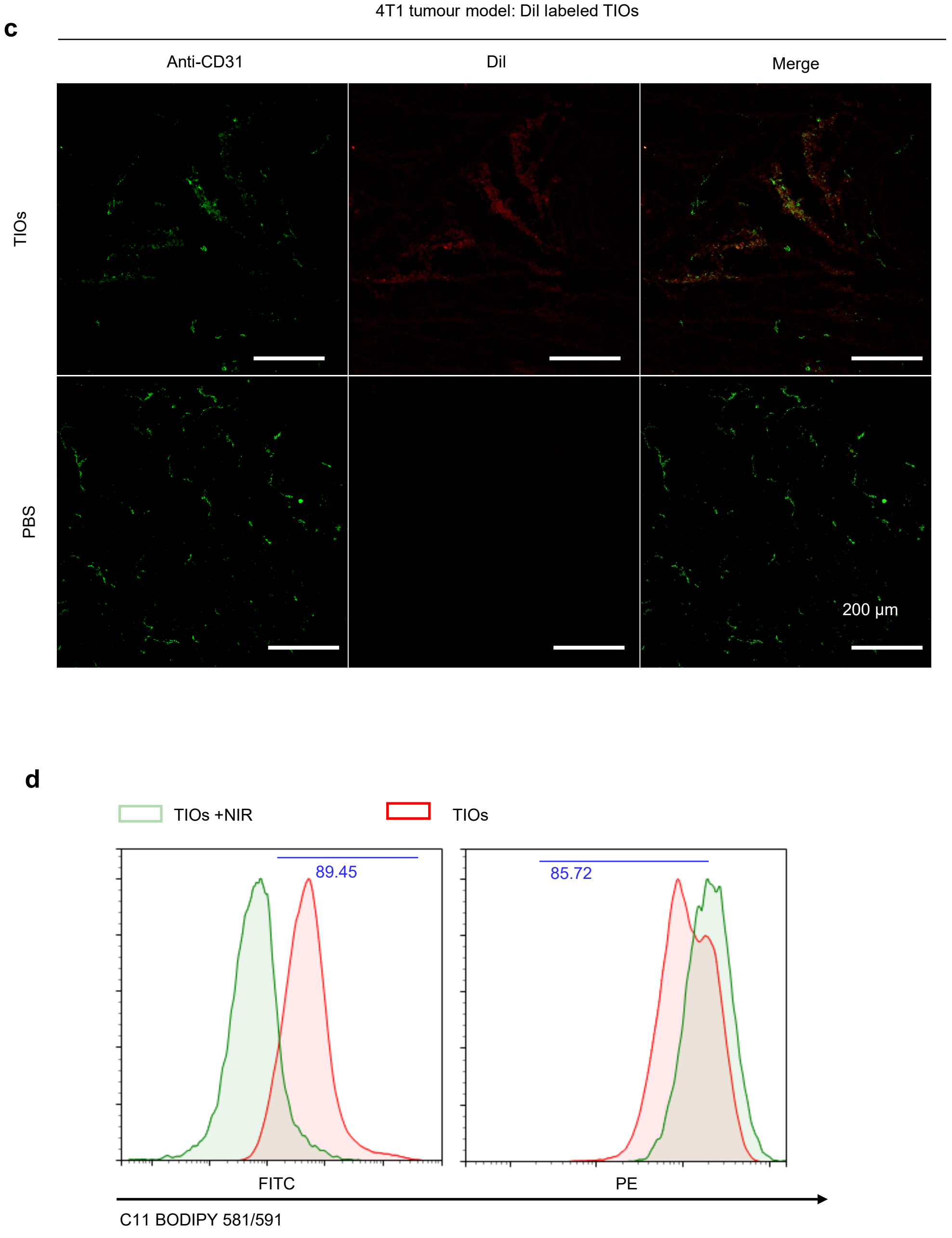

Supplementary Figure 9

Supplementary Figure 9. Biodistribution of RSL3@azo-Ce6-mSiO$_2$-UCNPs in 4T1 mice and tumour penetration of TIOs. a, Representative fluorescence ratio of tumour to liver at the indicated time points showed the biodistribution of DiI-labeled RSL3@azo-Ce6-mSiO2-UCNPs (370 μg/mouse) after intravenously injected to 4T1 tumour bearing mice. **b,** Average fluorescence intensity of DiI-labeled RSL3@azo-Ce6-mSiO$_2$-UCNPs (370 μg/mouse) in corresponding organs of indicated tumour models at a series of time points. **c**, Infiltration of tumour vasculature by DiI-labelled TIOs. Tumour microvessels are labelled by CD31 (green). Data in **b** are shown as mean ± s.d. (n = 3 independent mice, two-tailed unpaired Student's t-test was performed). **d**, Flow cytometry analysis of BODIPY fluorescence in tumors treated with TIOs+NIR or TIOs alone. Data shown are representative of two (**a-b**) or three (**c-d**) independent experiments. Source data are provided as a Source Data file.

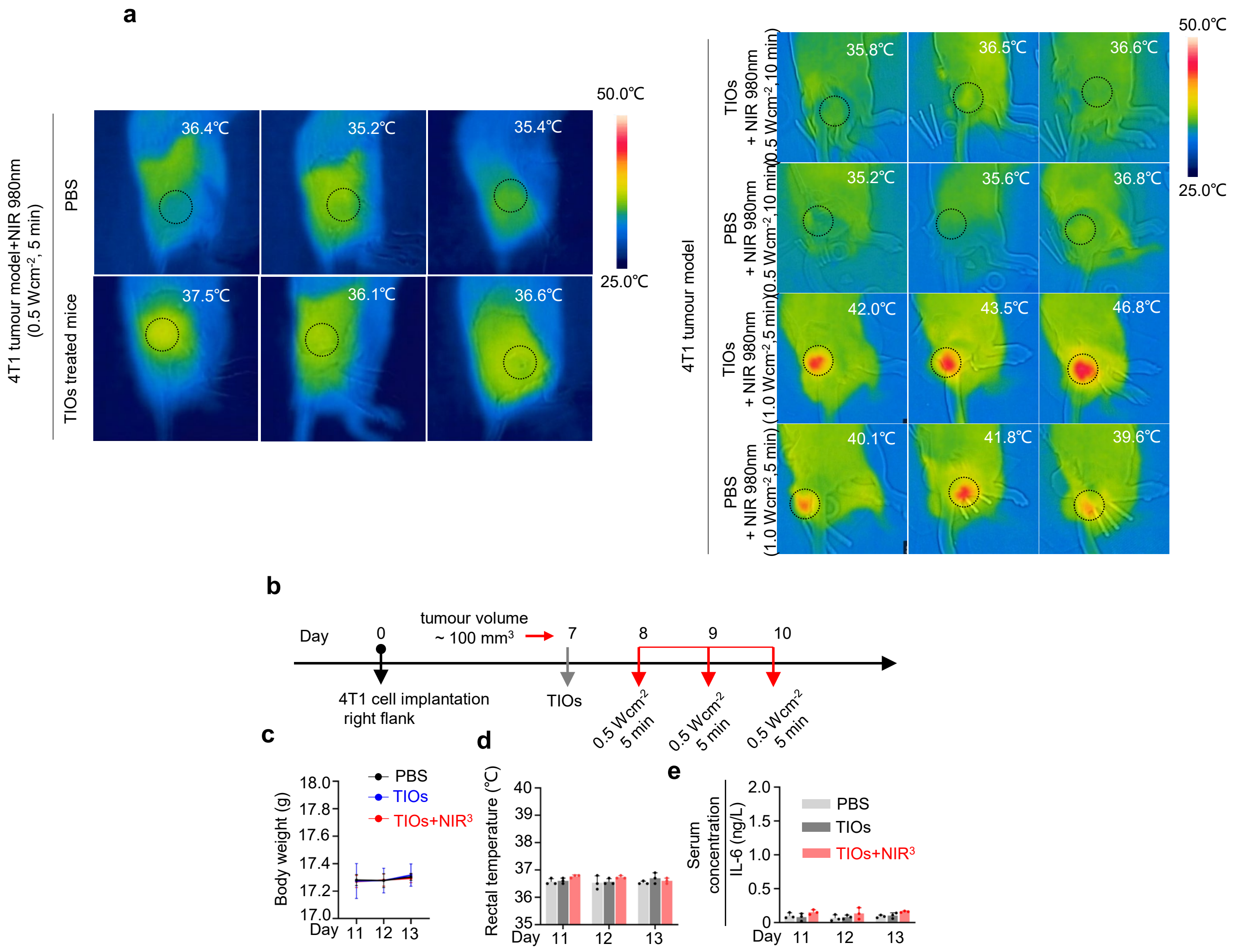

Supplementary Figure 10

Supplementary Figure 10. Analysis of photothermal effects and potential inflammatory effects of TIOs. a, The whole-body thermal imaging of subcutaneous 4T1 tumour-bearing BALB/c female mice treated with TIOs (5 x 10^6 units/mouse), or PBS after NIR 980nm (0.5 Wcm^{-2} or 1.0 Wcm^{-2}) for 5 min or 10 min. Images were obtained using a thermal camera. n = 3 mice for indicated groups. **b-e**, Inflammatory effects investigation of TIOs+NIR^3 treated 4T1 tumour-bearing mice. **b,** TIOs+NIR^3 treatment scheme in BALB/c mice implanted subcutaneously with 4T1 cells (3 mice per group). The tumour-bearing mice were intravenously injected with TIOs (iv, 5 x 10^6 units/mouse) and then treated with NIR light or not (980 nm, 0.5 W cm^{-2}, 5 min). **c**, The body weight of mice was measured at the indicated time. **d,** The rectal temperature of mice was measured at the indicated time. **e**, The levels of IL-6 in serum of mice were measured by ELISA at the indicated time. Data (**c-e**) are shown as mean ± s.e.m (n = 3 mice for indicated groups, two-tailed unpaired Student's t-test was performed). Data shown are representative of three independent experiments. Source data are provided as a Source Data file.

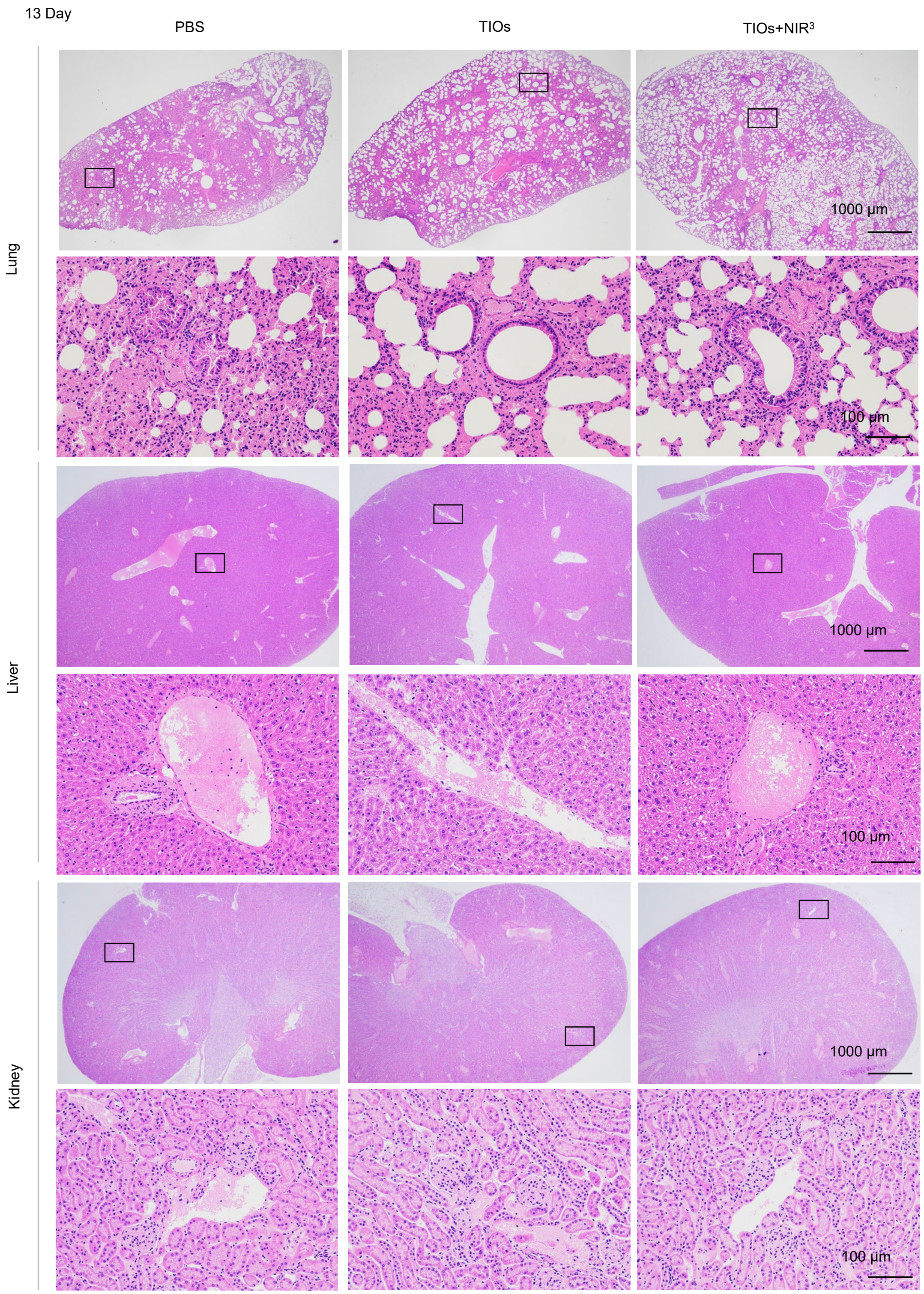

Supplementary Figure 11

Supplementary Figure 11. H & E analysis on day 13. Representative haematoxylin and eosin (H & E) staining of lung, liver and kidney tissues from mice which were collected on Day 13 post-treatments. Mice treated with PBS, TIOs, TIOs+NIR^3. Three mice per group. Data shown are representative of two independent experiments.

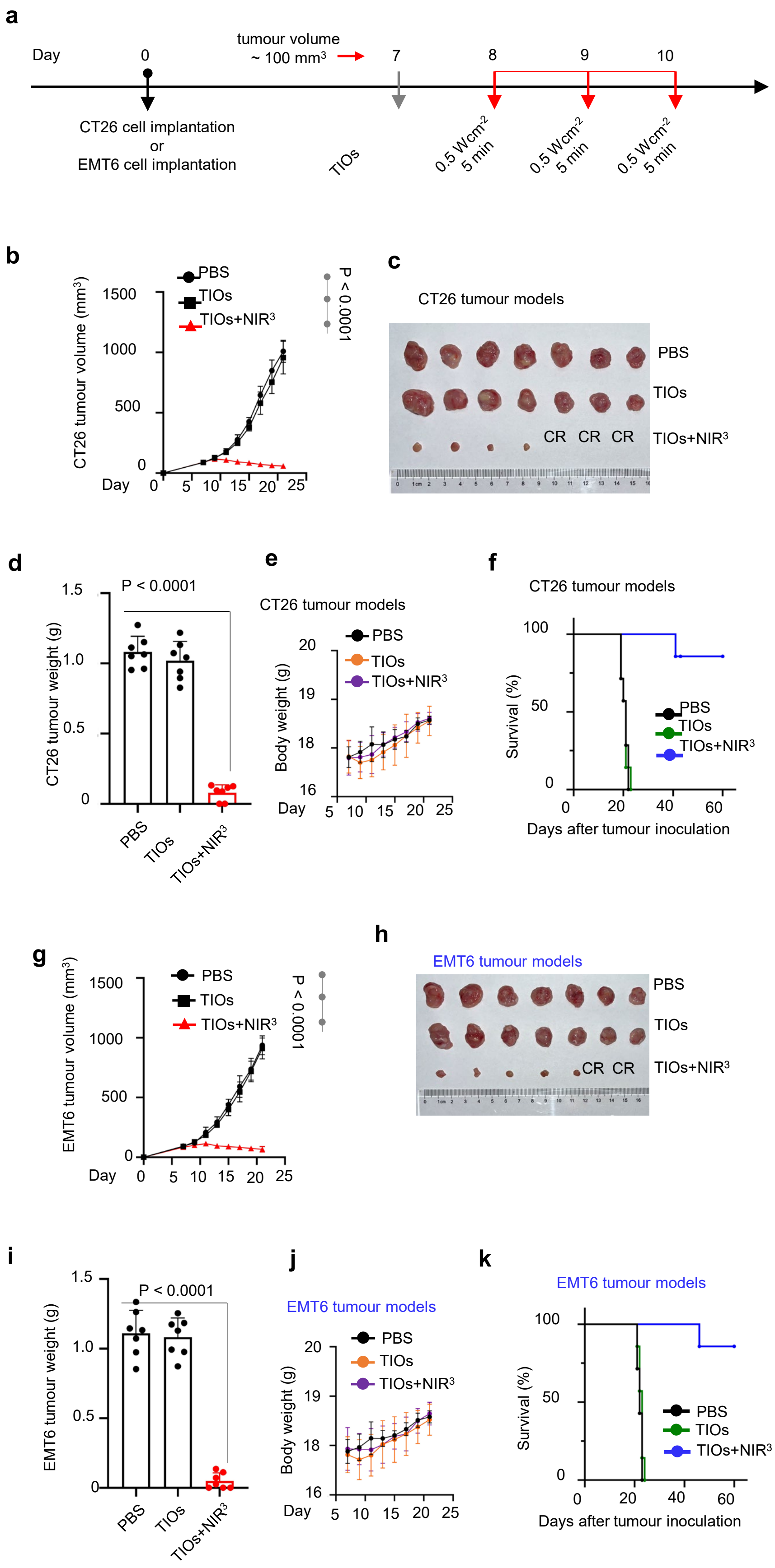

Supplementary Figure 12

Supplementary Figure 12. Antitumour effects in CT26 and EMT6 tumour models. a, The treatment scheme. **b-f**, The TIOs mediated antitumour effect in CT26 tumours. n = 7 mice. **b**, The tumour volume. **c**, The tumour images at day 25. **d**, The tumour weight at day 25. **e**, The body weight for 25 days. **f**, The survival curves for 60 days. **g-k**, The TIOs mediated antitumour effect in EMT6 tumours. n = 7 mice. **g**, The tumour volume. **h**, The tumour images at day 25. **i**, The tumour weight at day 25. **j**, The body weight for 25 days. **k**, The survival curves for 60 days. Data (**b, d-e, g, i-j**) are shown as mean ± s.e.m (two-tailed unpaired Student's t-test was performed). Data shown are representative of two independent experiments. Source data are provided as a Source Data file.

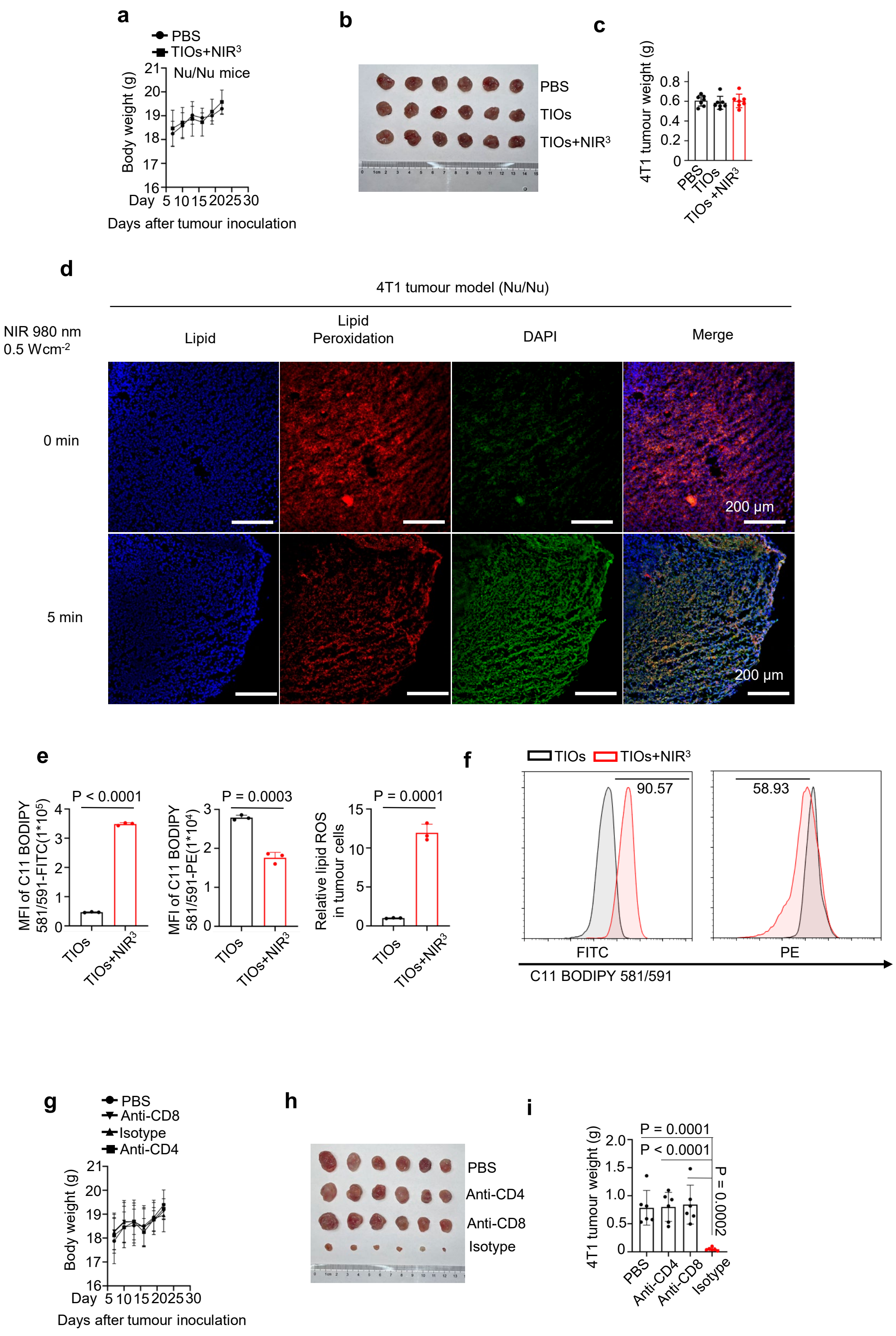

Supplementary Figure 13

Supplementary Figure 13. The TIOs mediated antitumour effects in Nude mice and T cell depletion mice. a-c, The antitumour effects in Nude mice (n = 6 mice). **a**, body weight of 4T1 tumour bearing mice. **b**, The image of 4T1 tumour bearing mice. **c**, The tumour weight at day 25. Data in **a, c** are shown as mean ± s.e.m (two-tailed unpaired Student's t-test was performed). **d-f**, lipids peroxidation in Nu/Nu mice. **d,** Representative fluorescence images of lipid peroxidation based on C11 581/591 BODIPY stained 4T1 tumours (Nu/Nu mice). TIOs+NIR treated mice (980 nm, 0.5 W cm^{-2}, 5 min) (n = 3 Nu/Nu mice) or TIOs alone treated mice (n = 3 Nu/Nu mice). Scale bars, 10 μm. **e,** Mean fluorescence intensities (MFI) of oxidized BODIPY (FITC channel) or, non-oxidized BODIPY (PE channel) and the relative lipid ROS in 4T1 tumours (Nu/Nu mice) with indicated treatments. The relative lipid ROS are caculated as the ratio of oxidized and non-oxidized BODIPY MFI. Tumour lipid peroxidation of 4T1 tumor-bearing mice treated with PBS, TIOs+NIR (980 nm, 0.5 Wcm^{-2}, 5 min) or TIOs alone, n = 3 Nu/Nu mice for indicated groups. **f,** Flow cytometry analysis of C11 581/591 BODIPY fluorescence in 4T1 tumours (Nu/Nu mice) treated with TIOs+NIR or TIOs alone. **g-i**, The antitumour effects in 4T1 tumour bearing Babl/c mice after T cell depletion mice. Anti-CD4 (n = 6 mice) or Anti-CD8 (n = 6 mice) antibodies or an isotype control (n = 6 mice) were intraperitoneally injected into BALB/c mice bearing 4T1 tumours before treatment. **g**, The body weight. **h**, The tumour image. **i**, The tumour weight after indicated treatments. Data in **e**, **g, i** are shown as mean ± s.e.m (two-tailed unpaired Student's t-test was performed). Data are representative of two (**a-c, g-i**) or three (**d-f**) independent experiments. Source data are provided as a Source Data file.

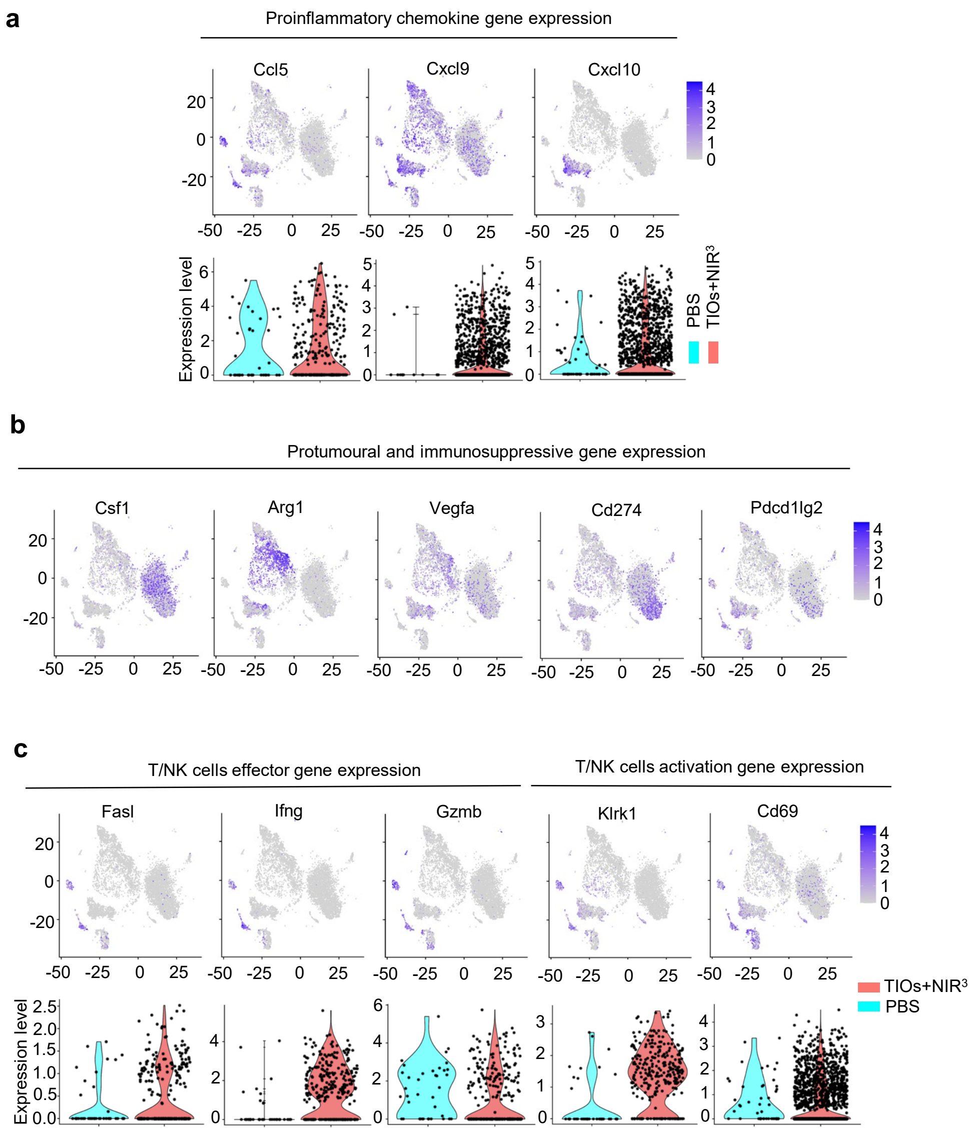

Supplementary Figure 14

Supplementary Figure 14. Expression levels of protumoural and immunosuppressive genes. **a**, Proinflammatory chemokine. **b**, Effector genes in immune cells. **c,** T, natural killer cell activation. Violin plots were used to compare the gene-expression levels in $CD45^{+}$ immune cells between 4T1 tumours treated with PBS or $TIOs+NIR^{3}$. Data are representative of two independent experiments.

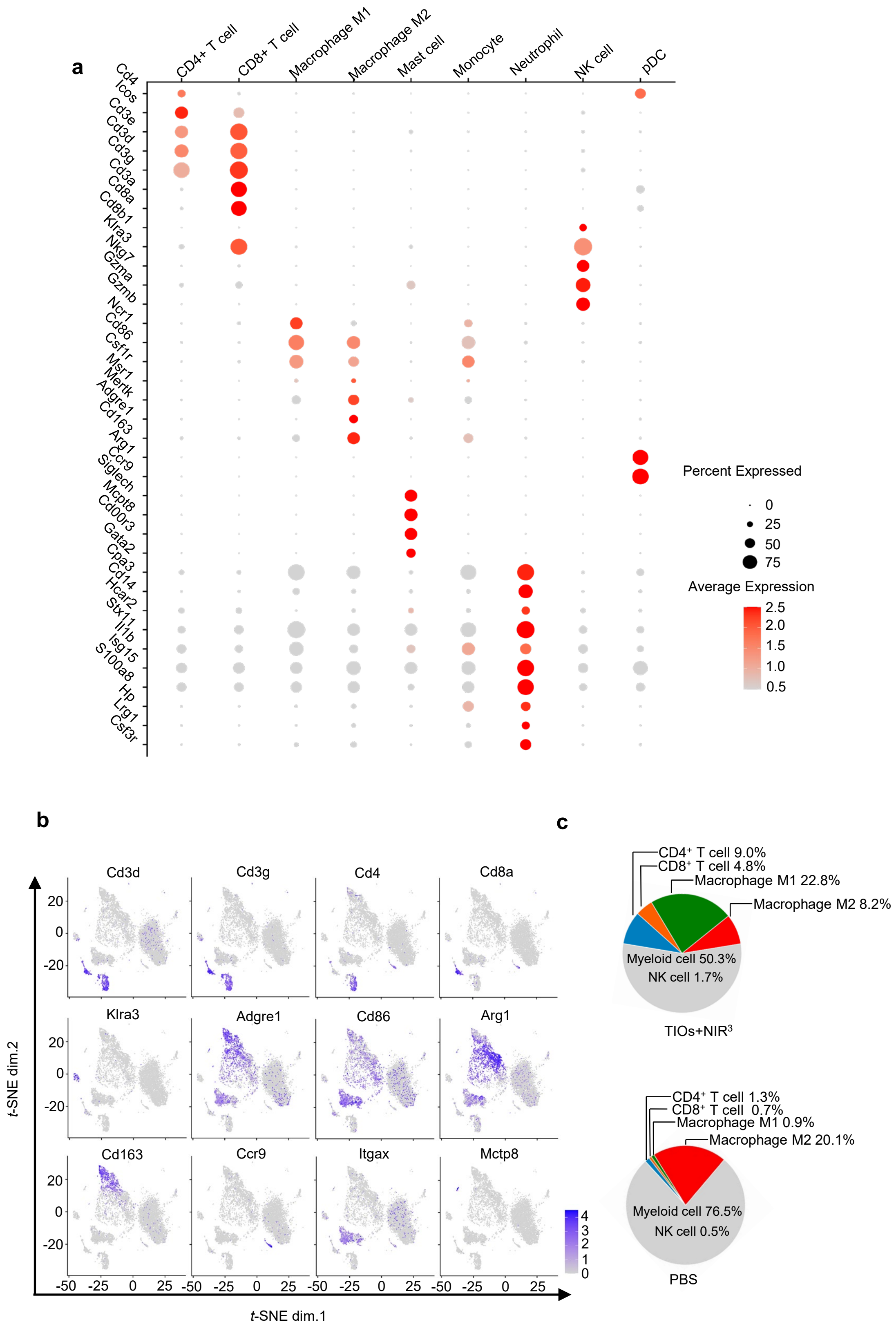

Supplementary Figure 15

Supplementary Figure 15. Tumour-infiltrating immune-cell-subtype analysis by single-cell RNA sequencing. a, Dot plots of selected markers from merged samples. Dot size indicates the proportion of cells in each cluster expressing. **b,** Signature gene-expression patterns for the corresponding cell clusters on the t-SNE plot. **c,** The relative frequencies of different clusters of tumour-infiltrating single $CD45^+$ immune cells of 4T1 tumours from mice treated with PBS (7,635 cells) or TIOs+NIR^3 (7,968 cells). Data are representative of two independent experiments.

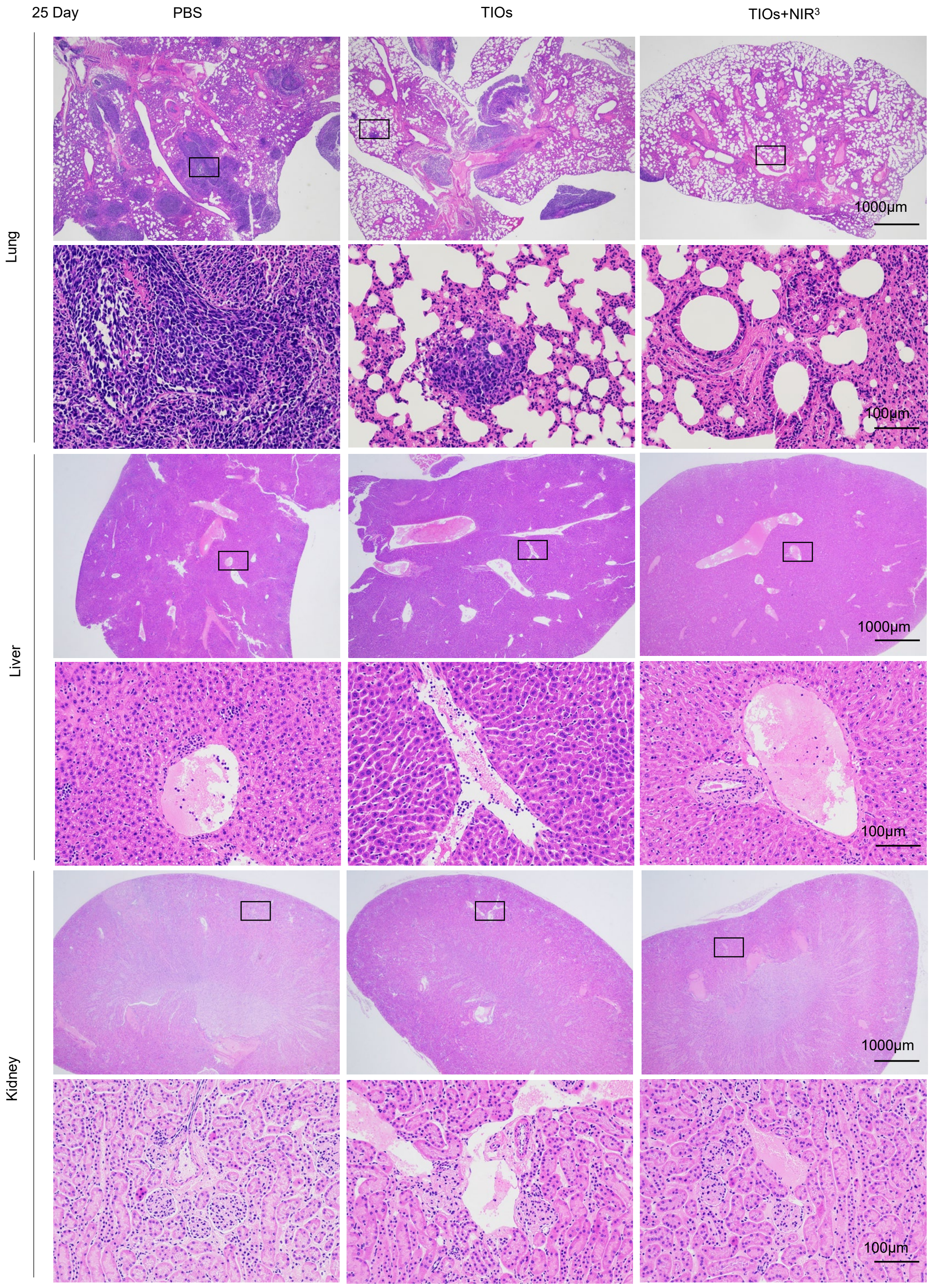

Supplementary Figure 16

Supplementary Figure 16. H & E analysis on day 25. Representative haematoxylin and eosin (H & E) staining of lung, liver and kidney tissues from mice which were collected on Day 13 post-treatments. Mice treated with PBS, TIOs, TIOs+NIR^3. Three mice per group. Data are representative of two independent experiments.

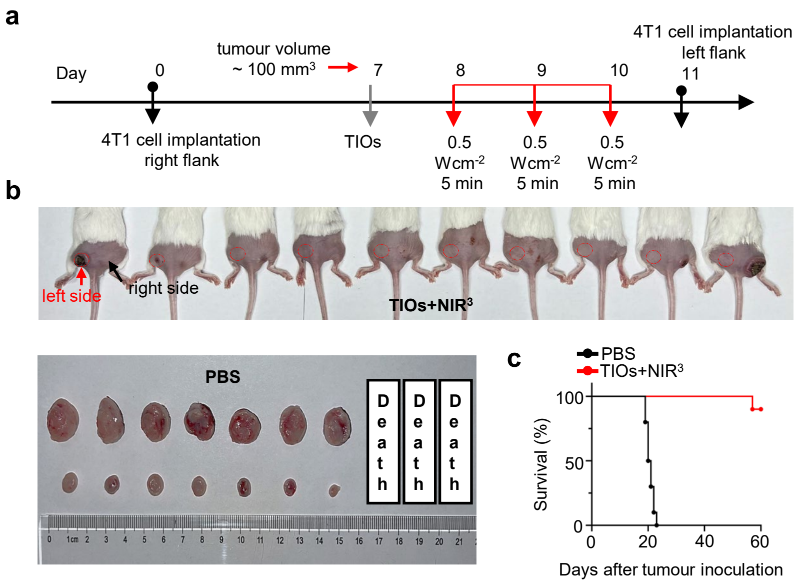

Supplementary Figure 17

Supplementary Figure 17. The antitumour immune memory effects. a-c, a, BALB/c mice were subcutaneously implanted with 4T1 (2 x 10^6 cells / mouse) in the right flanks at day 0. The tumour-bearing mice were intravenously injected (iv) with TIOs (5$\times$ 10^6 units / mouse) on day 7 and then treated with NIR light (980 nm, 0.5 W cm^{-2}, 5 min) at days 8 and 9,10, respectively. On day 11, the TIOs+NIR^3-treated mice were implanted subcutaneously again with 4T1 cells (5 x 10^5 cells /mouse) in the left flanks. **b,** Photograph of tumours of mice on day 60 (**top**, n = 10 mice for TIOs+NIR^3) and day 21 (**bottom**, n = 10 mice for PBS). **c,** Survival curves of 4T1 tumour-bearing mice (8 mice per group, log-rank test; $P < 0.0001$). Data are representative of two independent experiments. Source data are provided as a Source Data file.

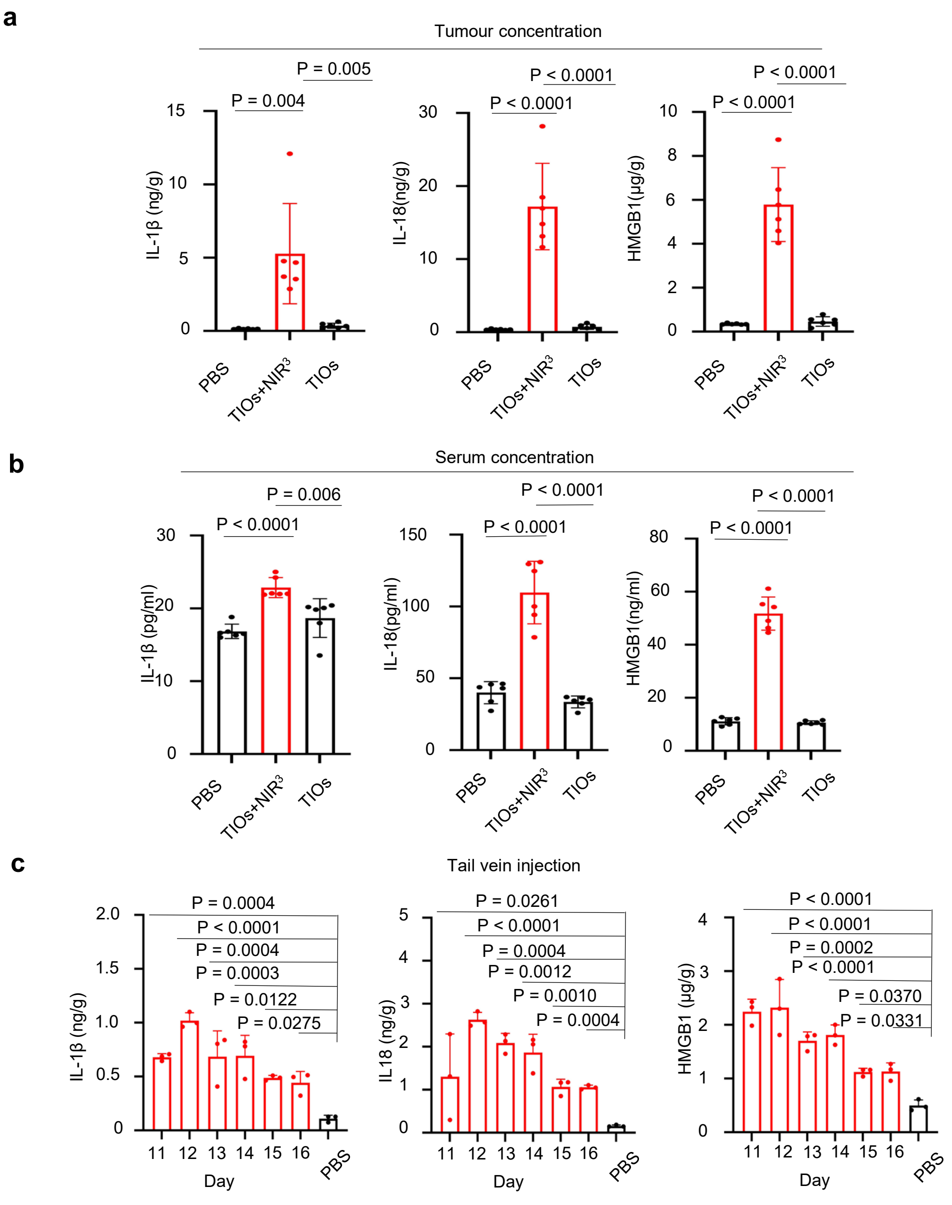

Supplementary Figure 18

Supplementary Figure 18. The cytokine release analysis. a-b, Enzyme-linked immunosorbent assay measurements of IL-18, IL-1β and HMGB1 concentrations in tumour homogenates (**a**) and the serum (**b**) of mice treated with PBS, TIOs or TIOs+NIR^3 (n = 6 mice, as shown in the figure for each group). **c,** A time-sequence cytokine release analysis. Enzyme-linked immunosorbent assay measurements of IL-18, IL-1β and HMGB1 concentrations in tumour homogenates in reduced oncolysis (n = 3 mice in indicated group). All data are shown as mean ± s.d. (two-tailed unpaired Student's *t* test). Data are representative of two independent experiments. Source data are provided as a Source Data file.

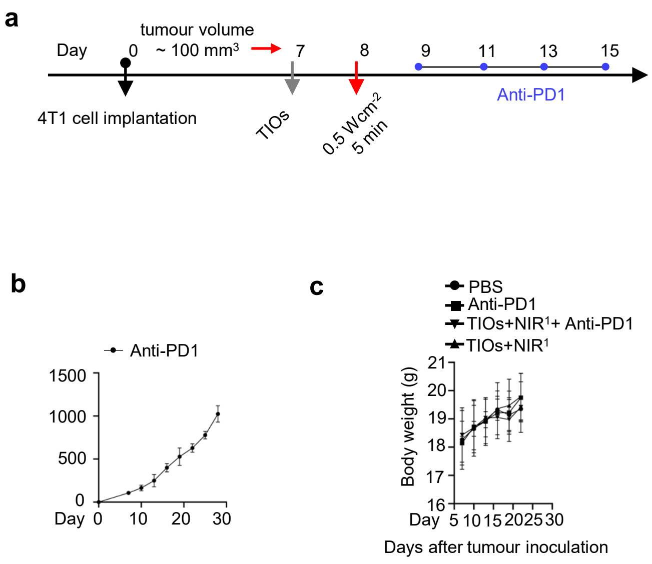

Supplementary Figure 19

Supplementary Figure 19. The reduced oncolysis synergized with anti-PD-1 therapy. a, The treatment scheme. **b**, The tumour volume of anti-PD-1 antibody treatment. **c**, The survival curves. Data are shown as mean ± s.e.m (n = 6 mice, two-tailed unpaired Student's t-test was performed). Data are representative of two independent experiments. Source data are provided as a Source Data file.

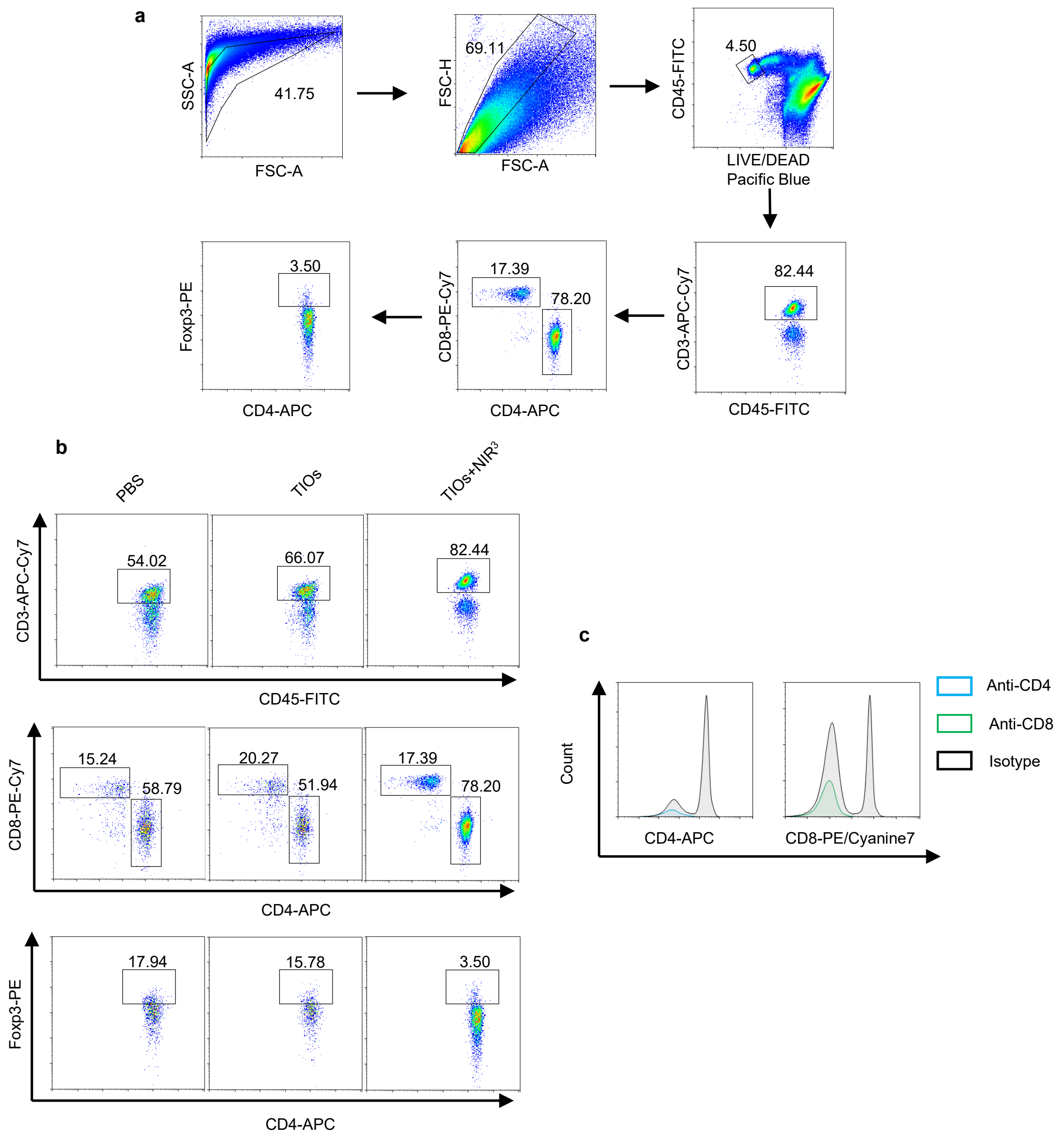

Supplementary Figure 20

Supplementary Figure 20. TIOs+NIR³ treatment stimulates inflammation and increases the tumour-infiltrating lymphocytes. a-b, Gating strategy (**a**) and representative flow-cytometry plots (**b**) for assessing 4T1 tumour-infiltrating $CD3^+$, $CD4^+$ or $CD8^+$ T cells or $FOXP3^+$ $CD4^+$ regulatory T cells following the indicated treatments. n = 8 mice for PBS, TIOs, and TIOs+NIR³. **c,** Flow-cytometry analysis of CD4+ or CD8+ T cells upon depletion by their corresponding antibody. Data shown are representative of two (**b-c**) independent experiments.

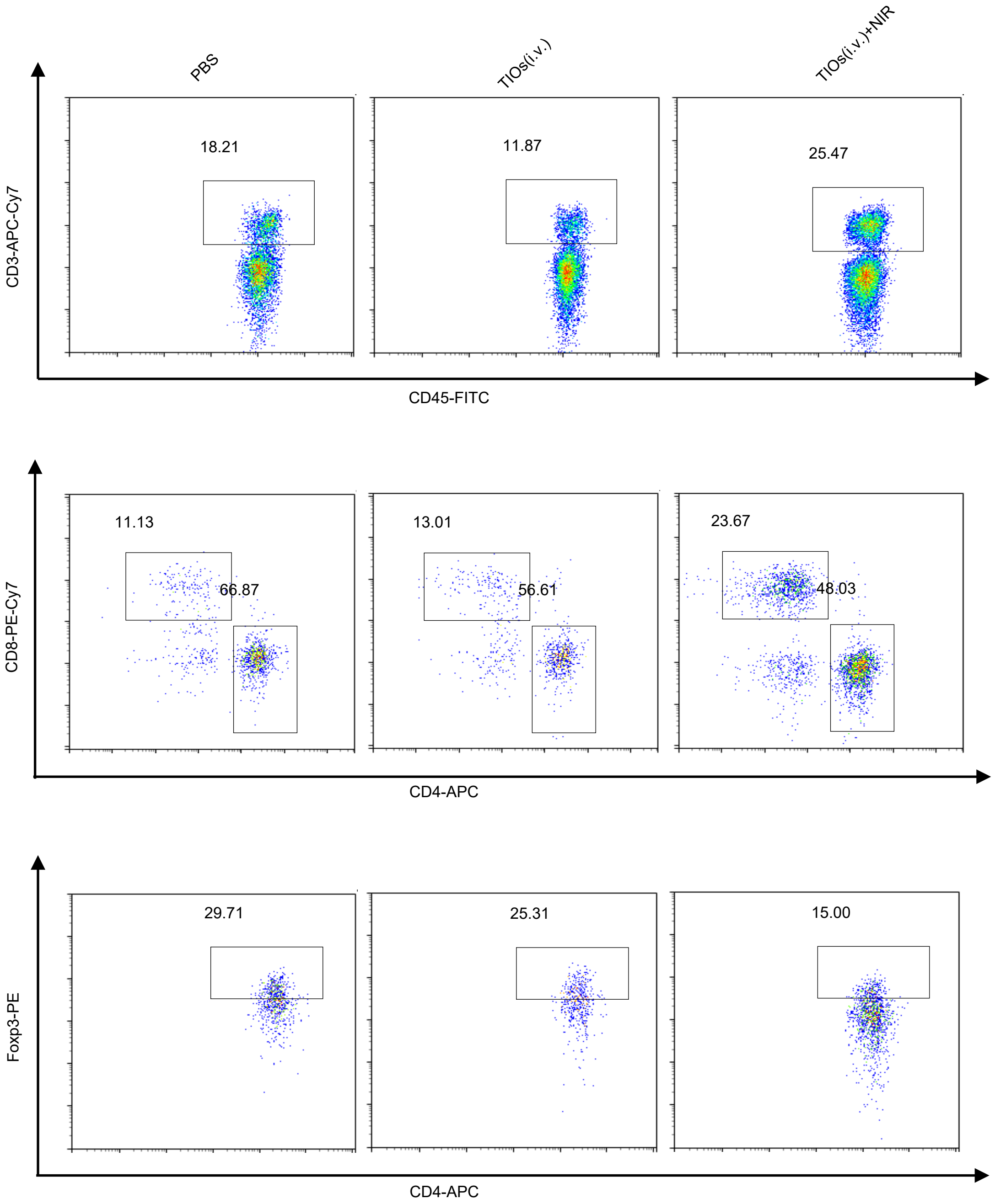

Supplementary Figure 21

Supplementary Figure 21. Representative flow-cytometry plots. For assessing 4T1 tumour-infiltrating $CD3^+$, $CD4^+$ or $CD8^+$ T cells or $FOXP3^+$ $CD4^+$ regulatory T cells following the indicated treatments. n = 6 mice for PBS, TIOs, and TIOs+NIR[3]. Data shown are representative of two independent experiments. Source data are provided as a Source Data file.

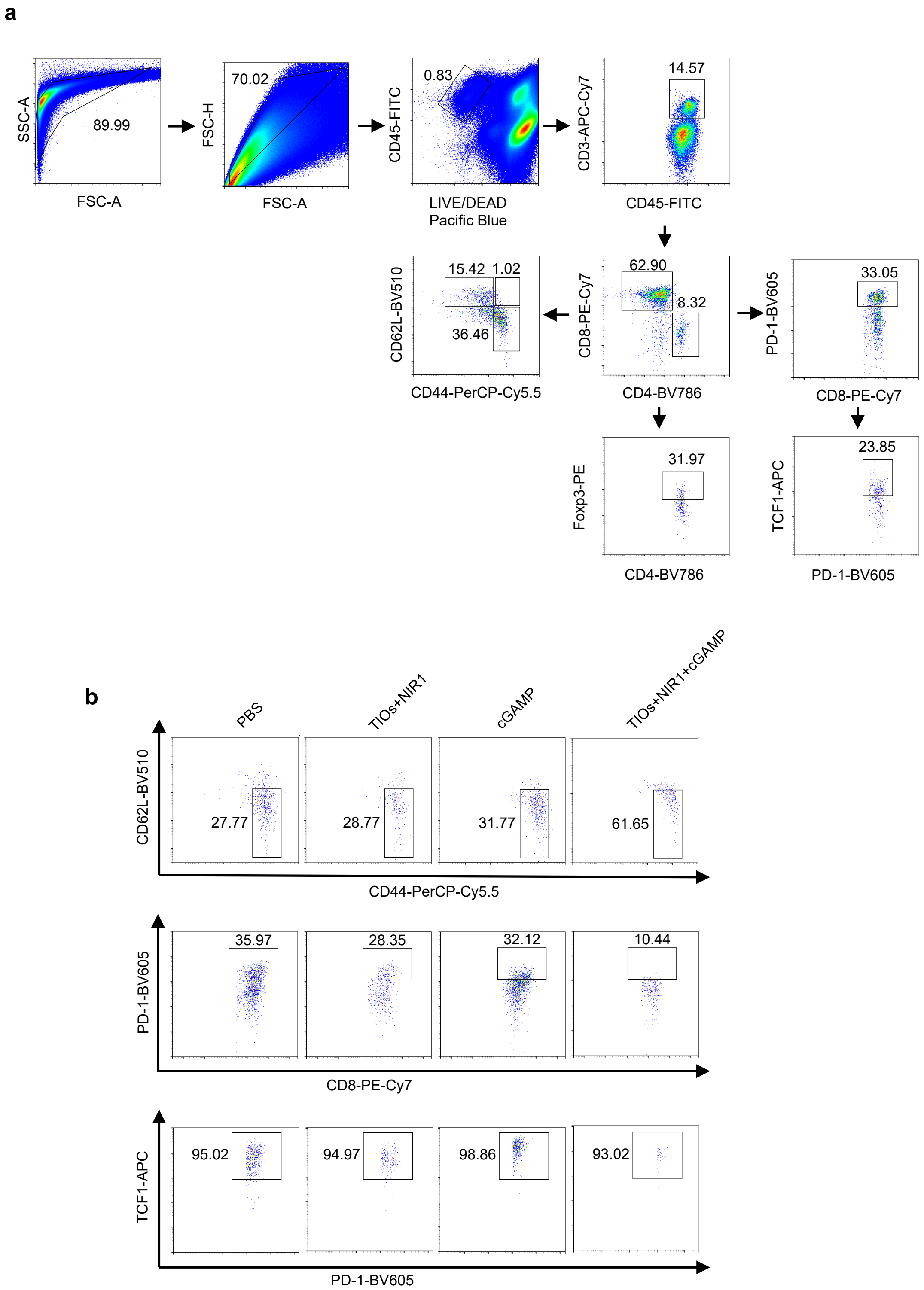

Supplementary Figure 22

Supplementary Figure 22. Gating strategy (**a**) and **representative flow-cytometry plots** (**b**) for assessing T cell exhaustion in reduced oncolysis and cGAMP synergizing. Data shown are representative of two (**b**) independent experiments.

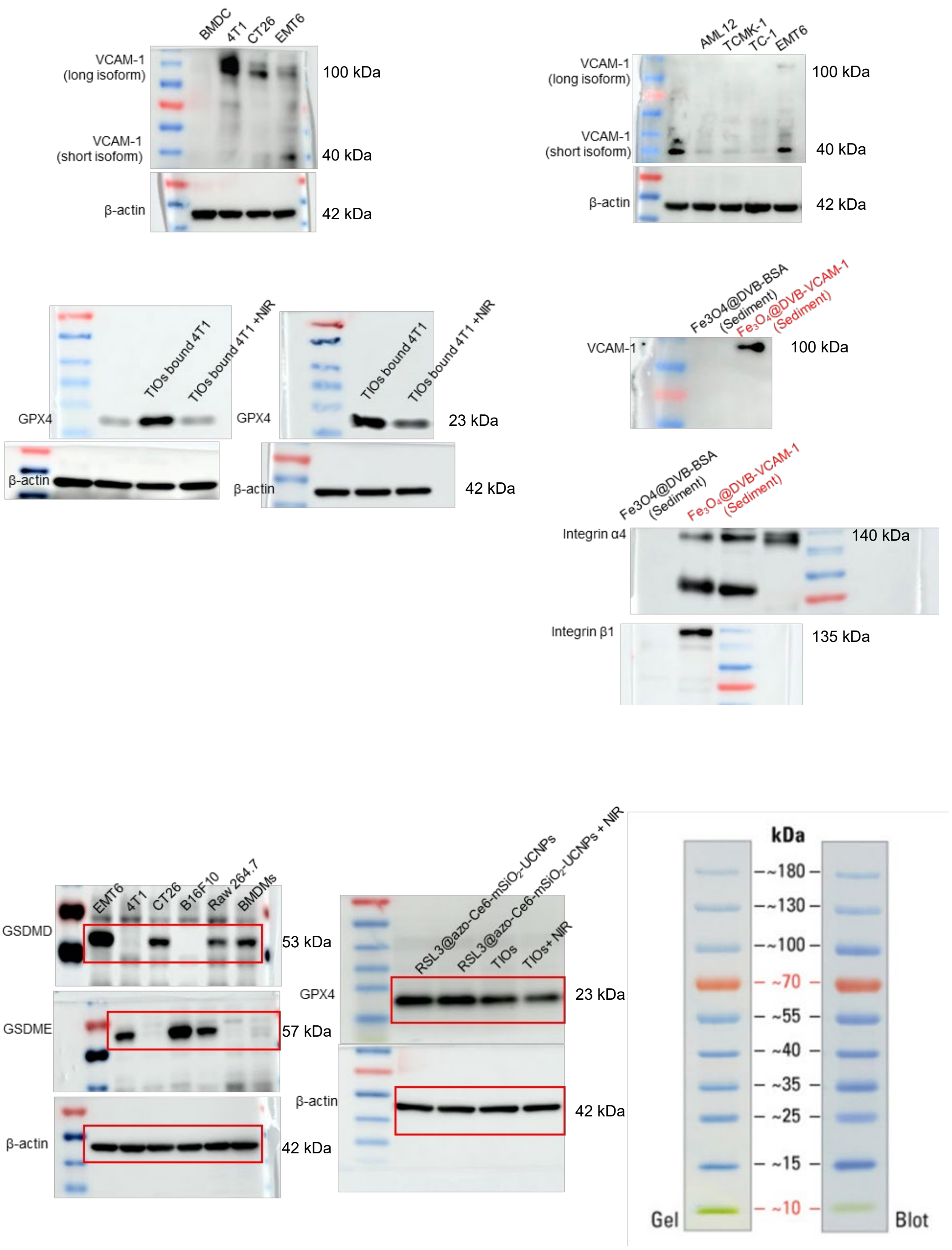

Supplementary Figure 23

Supplementary Figure 23. Full uncropped scans of any cropped gel/blot images.
