# Supplementary material for: A cytotoxic T cell inspired oncolytic nanosystem promotes lytic cell death by lipid peroxidation and elicits antitumor immune responses
Source: Nat Commun. 2023 Sep 6;14:5456. doi: 10.1038/s41467-023-41335-1 (PMC10482857; doi:10.1038/s41467-023-41335-1)
Supplement: Supplementary file 3 — Description of Additional Supplementary Files [file 41467_2023_41335_MOESM3_ESM.pdf]

Description of Additional Supplementary Files

Title: **Supplementary Movie 1. TIOs bound 4T1 with NIR**

Description: 4T1 cells were bound with TIOs, when irradiated with NIR light, 4T1 cells showed blowing bubbles and swelling.

Title: **Supplementary Movie 2. TIOs bound 4T1 without NIR**

Description: 4T1 cells were bound with TIOs in dark, 4T1 cells kept viablity and normal morphology.

Title: **Supplementary Movie 3. TIOs bound CT26 with NIR**

Description: CT26 cells were bound with TIOs, when irradiated with NIR light, CT26 cells showed blowing bubbles and swelling.

Title: **Supplementary Movie 4. TIOs bound CT26 without NIR**

Description: CT26 cells were bound with TIOs in dark, CT26 cells kept viablity and normal morphology.

Title: **Supplementary Movie 5. TIOs bound EMT6 with NIR**

Description: EMT6 cells were bound with TIOs, when irradiated with NIR light, EMT6 cells showed blowing bubbles and swelling.

Title: **Supplementary Movie 6. TIOs bound EMT6 without NIR**

Description: EMT6 cells were bound with TIOs in dark, EMT6 cells kept viablity and normal morphology.
