# Supplementary material for: A cytotoxic T cell inspired oncolytic nanosystem promotes lytic cell death by lipid peroxidation and elicits antitumor immune responses
Source: Nat Commun. 2023 Sep 6;14:5456. doi: 10.1038/s41467-023-41335-1 (PMC10482857; doi:10.1038/s41467-023-41335-1)
Supplement: Supplementary file 10 — Reporting Summary [file 41467_2023_41335_MOESM10_ESM.pdf]

nature portfolio

Corresponding author(s): Qinyang Wang

Last updated by author(s): Aug 14, 2023

Reporting Summary

Nature Portfolio wishes to improve the reproducibility of the work that we publish. This form provides structure for consistency and transparency in reporting. For further information on Nature Portfolio policies, see our Editorial Policies and the Editorial Policy Checklist.

Statistics

For all statistical analyses, confirm that the following items are present in the figure legend, table legend, main text, or Methods section.

n/a	Confirmed	
☐	☒	The exact sample size (*n*) for each experimental group/condition, given as a discrete number and unit of measurement
☐	☒	A statement on whether measurements were taken from distinct samples or whether the same sample was measured repeatedly
☐	☒	The statistical test(s) used AND whether they are one- or two-sided<br>*Only common tests should be described solely by name; describe more complex techniques in the Methods section.*
☒	☐	A description of all covariates tested
☐	☒	A description of any assumptions or corrections, such as tests of normality and adjustment for multiple comparisons
☐	☒	A full description of the statistical parameters including central tendency (e.g. means) or other basic estimates (e.g. regression coefficient) AND variation (e.g. standard deviation) or associated estimates of uncertainty (e.g. confidence intervals)
☐	☒	For null hypothesis testing, the test statistic (e.g. *F*, *t*, *r*) with confidence intervals, effect sizes, degrees of freedom and *P* value noted<br>*Give P values as exact values whenever suitable.*
☒	☐	For Bayesian analysis, information on the choice of priors and Markov chain Monte Carlo settings
☒	☐	For hierarchical and complex designs, identification of the appropriate level for tests and full reporting of outcomes
☒	☐	Estimates of effect sizes (e.g. Cohen's *d*, Pearson's *r*), indicating how they were calculated

Our web collection on statistics for biologists contains articles on many of the points above.

Software and code

Policy information about availability of computer code

Data collection	Flow cytometry data were collected via AgilentFACS (Novocyte);<br>CCK8 , LDH release and calcein-AM release data were collected via multifunctional microplate reader (Tecan Spark);<br>The size and zeta potential data were collected via DLS (Malvern Zetasizer Nano S90);<br>In-vivo Imaging were collected via in vivo imaging system (Analytik UVP iBox®Scientia™).
Data analysis	All statistical analyses were performed on the GraphPad Prism software package (PRISM 8.0; GraphPad Prism Software);<br>All the flow cytometry data were processed using FlowJo (version 10.4.0, Becton, Dickinson & Company);<br>Images and videos were processed and analyzed by Olympus FV31S software;<br>In-vivo Imaging data were processed using VistionWorks (version 9.1.20063.7760).

For manuscripts utilizing custom algorithms or software that are central to the research but not yet described in published literature, software must be made available to editors and reviewers. We strongly encourage code deposition in a community repository (e.g. GitHub). See the Nature Portfolio guidelines for submitting code & software for further information.

Data

Policy information about availability of data

All manuscripts must include a data availability statement. This statement should provide the following information, where applicable:

- Accession codes, unique identifiers, or web links for publicly available datasets
- A description of any restrictions on data availability
- For clinical datasets or third party data, please ensure that the statement adheres to our policy

All data generated or analysed during this study are included in this manuscript and its supplementary information. Source data and uncropped blot images are provided with the paper.

Human research participants

Policy information about studies involving human research participants and Sex and Gender in Research.

Reporting on sex and gender	*Use the terms sex (biological attribute) and gender (shaped by social and cultural circumstances) carefully in order to avoid confusing both terms. Indicate if findings apply to only one sex or gender; describe whether sex and gender were considered in study design whether sex and/or gender was determined based on self-reporting or assigned and methods used. Provide in the source data disaggregated sex and gender data where this information has been collected, and consent has been obtained for sharing of individual-level data; provide overall numbers in this Reporting Summary. Please state if this information has not been collected. Report sex- and gender-based analyses where performed, justify reasons for lack of sex- and gender-based analysis.*
Population characteristics	*Describe the covariate-relevant population characteristics of the human research participants (e.g. age, genotypic information, past and current diagnosis and treatment categories). If you filled out the behavioural & social sciences study design questions and have nothing to add here, write "See above."*
Recruitment	*Describe how participants were recruited. Outline any potential self-selection bias or other biases that may be present and how these are likely to impact results.*
Ethics oversight	*Identify the organization(s) that approved the study protocol.*

Note that full information on the approval of the study protocol must also be provided in the manuscript.

Field-specific reporting

Please select the one below that is the best fit for your research. If you are not sure, read the appropriate sections before making your selection.

☒ Life sciences ☐ Behavioural & social sciences ☐ Ecological, evolutionary & environmental sciences

For a reference copy of the document with all sections, see nature.com/documents/nr-reporting-summary-flat.pdf

Life sciences study design

All studies must disclose on these points even when the disclosure is negative.

Sample size	No statistical methods were used to predetermine the sample sizes. It is impossible to predict the magnitude of experimental variation between animals based on our current knowledge. The group sizes (at least three animals per treatment group) represents the minimum number animals needed to reach statistical significance ($p< 0.05$) between experimental groups.
Data exclusions	No data were excluded.
Replication	Experiment were repeated and experimental findings were reproducible. Details of experimental replicates are given in the figure legends. All reported attempts at replication were successful.
Randomization	All experimental samples or models were allocated randomly to each group.
Blinding	No blinding was done in this study. Most of the studies contained multiple steps (including the material preparation, mouse tumor treatment, and so on) and the scientists must keep careful track of conditions. It would be exceedingly difficult to blind such studies.

Behavioural & social sciences study design

All studies must disclose on these points even when the disclosure is negative.

Study description	*Briefly describe the study type including whether data are quantitative, qualitative, or mixed-methods (e.g. qualitative cross-sectional, quantitative experimental, mixed-methods case study).*
Research sample	*State the research sample (e.g. Harvard university undergraduates, villagers in rural India) and provide relevant demographic information (e.g. age, sex) and indicate whether the sample is representative. Provide a rationale for the study sample chosen. For studies involving existing datasets, please describe the dataset and source.*
Sampling strategy	*Describe the sampling procedure (e.g. random, snowball, stratified, convenience). Describe the statistical methods that were used to predetermine sample size OR if no sample-size calculation was performed, describe how sample sizes were chosen and provide a rationale for why these sample sizes are sufficient. For qualitative data, please indicate whether data saturation was considered, and what criteria were used to decide that no further sampling was needed.*
Data collection	*Provide details about the data collection procedure, including the instruments or devices used to record the data (e.g. pen and paper, computer, eye tracker, video or audio equipment) whether anyone was present besides the participant(s) and the researcher, and whether the researcher was blind to experimental condition and/or the study hypothesis during data collection.*
Timing	*Indicate the start and stop dates of data collection. If there is a gap between collection periods, state the dates for each sample cohort.*
Data exclusions	*If no data were excluded from the analyses, state so OR if data were excluded, provide the exact number of exclusions and the rationale behind them, indicating whether exclusion criteria were pre-established.*
Non-participation	*State how many participants dropped out/declined participation and the reason(s) given OR provide response rate OR state that no participants dropped out/declined participation.*
Randomization	*If participants were not allocated into experimental groups, state so OR describe how participants were allocated to groups, and if allocation was not random, describe how covariates were controlled.*

Ecological, evolutionary & environmental sciences study design

All studies must disclose on these points even when the disclosure is negative.

Study description	*Briefly describe the study. For quantitative data include treatment factors and interactions, design structure (e.g. factorial, nested, hierarchical), nature and number of experimental units and replicates.*
Research sample	*Describe the research sample (e.g. a group of tagged Passer domesticus, all Stenocereus thurberi within Organ Pipe Cactus National Monument), and provide a rationale for the sample choice. When relevant, describe the organism taxa, source, sex, age range and any manipulations. State what population the sample is meant to represent when applicable. For studies involving existing datasets, describe the data and its source.*
Sampling strategy	*Note the sampling procedure. Describe the statistical methods that were used to predetermine sample size OR if no sample-size calculation was performed, describe how sample sizes were chosen and provide a rationale for why these sample sizes are sufficient.*
Data collection	*Describe the data collection procedure, including who recorded the data and how.*
Timing and spatial scale	*Indicate the start and stop dates of data collection, noting the frequency and periodicity of sampling and providing a rationale for these choices. If there is a gap between collection periods, state the dates for each sample cohort. Specify the spatial scale from which the data are taken*
Data exclusions	*If no data were excluded from the analyses, state so OR if data were excluded, describe the exclusions and the rationale behind them, indicating whether exclusion criteria were pre-established.*
Reproducibility	*Describe the measures taken to verify the reproducibility of experimental findings. For each experiment, note whether any attempts to repeat the experiment failed OR state that all attempts to repeat the experiment were successful.*
Randomization	*Describe how samples/organisms/participants were allocated into groups. If allocation was not random, describe how covariates were controlled. If this is not relevant to your study, explain why.*
Blinding	*Describe the extent of blinding used during data acquisition and analysis. If blinding was not possible, describe why OR explain why blinding was not relevant to your study.*

Did the study involve field work? ☐ Yes ☐ No

Field work, collection and transport

Field conditions	*Describe the study conditions for field work, providing relevant parameters (e.g. temperature, rainfall).*
Location	*State the location of the sampling or experiment, providing relevant parameters (e.g. latitude and longitude, elevation, water depth).*
Access & import/export	*Describe the efforts you have made to access habitats and to collect and import/export your samples in a responsible manner and in compliance with local, national and international laws, noting any permits that were obtained (give the name of the issuing authority, the date of issue, and any identifying information).*
Disturbance	*Describe any disturbance caused by the study and how it was minimized.*

Reporting for specific materials, systems and methods

We require information from authors about some types of materials, experimental systems and methods used in many studies. Here, indicate whether each material, system or method listed is relevant to your study. If you are not sure if a list item applies to your research, read the appropriate section before selecting a response.

Materials & experimental systems

n/a	Involved in the study	
☐	☒	Antibodies
☐	☒	Eukaryotic cell lines
☒	☐	Palaeontology and archaeology
☐	☒	Animals and other organisms
☒	☐	Clinical data
☒	☐	Dual use research of concern

Methods

n/a	Involved in the study	
☒	☐	ChIP-seq
☐	☒	Flow cytometry
☒	☐	MRI-based neuroimaging

Antibodies

Antibodies used

Anti-glutathione peroxidase 4 (ab125066) was purchased from Abcam. Integrin α4 (D2E1) XP® rabbit mAb (CST8440), Integrin β1 (D6S1 W) rabbit mAb (CST34971), VCAM-1 (D8U5 V) rabbit mAb (CST39036), SIRPα/SHPS1 (D6I3 M) rabbit mAb (CST13379), anti-rabbit IgG (H+L), F(ab')2 fragment (Alexa Fluor® 488 conjugate) (CST4412), anti-rat IgG (H+L), (Alexa Fluor® 488 Conjugate) (CST4416), anti-rabbit IgG, HRP-linked Antibody(CST7074), anti-β-actin (CST8457) antibodies were purchased from Cell Signaling Technology. The CD47 rabbit mAb (A1838) was purchased from ABclonal. Purified anti-mouse CD31 Antibody, Purified anti-mouse Siglec-G Antibody, FITC anti-mouse CD45 antibody-S18009F, APC/Cyanine7 anti-mouse CD3-17A2, APC anti-mouse CD4-RM4-4, PE/Cyanine7 anti-mouse CD8a-53-6.7, Brilliant Violet 605 anti-mouse CD279 (PD-1) and Brilliant Violet 785™ anti-mouse CD274 (B7-H1, PD-L1) were purchased from BioLegend. Ms CD4 BV786 GK1.5, Ms CD44 PerCP-Cy5.5 IM7, Ms CD62L BV510 MEL-14, TCF-7/TCF-1 Alexa 647 S33-966 were purchased from BD Biosciences. CD24 Monoclonal Antibody, PE FOXP3 monoclonal antibody (FJK-16s) was obtained from Invitrogen. The InVivoMAb anti-mouse PD-1 antibody (clone J43) used to treat 4T1 tumours was obtained from BioXcell. For immune cell depletion, anti-mouse CD4 (clone GK1.5), anti-mouse CD8 (clone YTS 169.4) antibodies were produced by BioXcell.

Validation

https://www.abcam.cn/glutathione-peroxidase-4-antibody-epncir144-ab125066.html
https://www.cellsignal.cn/products/primary-antibodies/integrin-a4-d2e1-xp-rabbit-mab/8440?site-search-type=Products&N=4294956287&Ntt=8440&fromPage=plp&_requestid=2557024
https://www.cellsignal.cn/products/primary-antibodies/integrin-b1-d6s1w-rabbit-mab/34971?site-search-type=Products&N=4294956287&Ntt=34971&fromPage=plp&_requestid=2557092
https://www.cellsignal.cn/products/primary-antibodies/vcam-1-d8u5v-rabbit-mab-mouse-specific/39036?site-search-type=Products&N=4294956287&Ntt=39036&fromPage=plp&_requestid=2557167
https://www.cellsignal.cn/products/primary-antibodies/sirpa-shps1-d6i3m-rabbit-mab/13379?site-search-type=Products&N=4294956287&Ntt=13379&fromPage=plp&_requestid=2557254
https://www.cellsignal.cn/products/secondary-antibodies/anti-rabbit-igg-h-l-f-ab-2-fragment-alexa-fluor-488-conjugate/4412?site-search-type=Products&N=4294956287&Ntt=4412&fromPage=plp&_requestid=2557319
https://www.cellsignal.cn/products/secondary-antibodies/anti-rat-igg-h-l-alexa-fluor-488-conjugate/4416?site-search-type=Products&N=4294956287&Ntt=4416&fromPage=plp&_requestid=510729
https://www.cellsignal.cn/products/secondary-antibodies/anti-rabbit-igg-hrp-linked-antibody/7074?site-search-type=Products&N=4294956287&Ntt=7074&fromPage=plp&_requestid=2556807
https://www.cellsignal.cn/products/primary-antibodies/b-actin-d6a8-rabbit-mab/8457?site-search-type=Products&N=4294956287&Ntt=8457&fromPage=plp&_requestid=2557431
https://abclonal.com.cn/catalog/A1838
https://www.biolegend.com/en-us/products/purified-anti-mouse-cd31-antibody-123
https://www.biolegend.com/en-us/products/purified-anti-mouse-siglec-g-antibody-20651
https://www.biolegend.com/en-us/products/fitc-anti-mouse-cd45-antibody-21256
https://www.biolegend.com/en-us/products/apc-cyanine7-anti-mouse-cd3-antibody-6068

https://www.biolegend.com/en-us/products/apc-anti-mouse-cd4-antibody-9224
https://www.biolegend.com/en-us/products/pe-cyanine7-anti-mouse-cd8a-antibody-1906
https://www.biolegend.com/en-us/products/brilliant-violet-605-anti-mouse-cd279-pd-1-antibody-7648
https://www.biolegend.com/en-us/products/brilliant-violet-785-anti-mouse-cd274-b7-h1-pd-l1-antibody-13497
https://www.bdbiosciences.com/zh-cn/products/reagents/flow-cytometry-reagents/research-reagents/single-color-antibodies-ruo/bv786-rat-anti-mouse-cd4.563331
https://www.bdbiosciences.com/zh-cn/products/reagents/flow-cytometry-reagents/research-reagents/single-color-antibodies-ruo/percp-cy-5-5-rat-anti-mouse-cd44.560570
https://www.bdbiosciences.com/zh-cn/products/reagents/flow-cytometry-reagents/research-reagents/single-color-antibodies-ruo/bv510-rat-anti-mouse-cd62l.563117
https://www.bdbiosciences.com/zh-cn/products/reagents/flow-cytometry-reagents/research-reagents/single-color-antibodies-ruo/alexa-fluor-647-mouse-anti-tcf-7-tcf-1.566693
https://www.thermofisher.cn/cn/zh/antibody/product/CD24-Antibody-clone-M1-69-Monoclonal/14-0242-82
https://www.thermofisher.cn/cn/zh/antibody/product/FOXP3-Antibody-clone-FJK-16s-Monoclonal/12-5773-82
https://bioxcell.com/invivomab-anti-mouse-pd-1-cd279-be0033-2
https://bioxcell.com/invivoplus-anti-mouse-cd4-bp0003-1
https://bioxcell.com/invivoplus-anti-mouse-cd8a-bp0117

Eukaryotic cell lines

Policy information about cell lines and Sex and Gender in Research

Cell line source(s)	4T1, CT26, EMT6,and Raw 264.7 cells were kindly provided by the Stem Cell Bank, Chinese Academy of Sciences. EMT6, TC-1, AML12 and TCMK-1 cells were obtained from the American Type Culture Collection (ATCC). Mouse BMDMs were differentiated in vitro from isolated bone marrow cells. Bone marrow cells collected from mouse femurs and tibias were incubated for 7 days in DMEM containing 20% FBS, 1% Pen Strep, and 10 ng/mL M-CSF (Sigma–Aldrich).
Authentication	Identity of the cell lines were frequently checked by their morphological features but have not been authenticated by the short tandem repeat (STR) profiling
Mycoplasma contamination	All cell lines were tested for mycoplasma contamination. No mycoplasma contamination was found.
Commonly misidentified lines (See ICLAC register)	No commonly misidentified cell lines are used in this study.

Palaeontology and Archaeology

Specimen provenance	*Provide provenance information for specimens and describe permits that were obtained for the work (including the name of the issuing authority, the date of issue, and any identifying information). Permits should encompass collection and, where applicable, export.*
Specimen deposition	*Indicate where the specimens have been deposited to permit free access by other researchers.*
Dating methods	*If new dates are provided, describe how they were obtained (e.g. collection, storage, sample pretreatment and measurement), where they were obtained (i.e. lab name), the calibration program and the protocol for quality assurance OR state that no new dates are provided.*

☐ Tick this box to confirm that the raw and calibrated dates are available in the paper or in Supplementary Information.

Ethics oversight	*Identify the organization(s) that approved or provided guidance on the study protocol, OR state that no ethical approval or guidance was required and explain why not.*

Note that full information on the approval of the study protocol must also be provided in the manuscript.

Animals and other research organisms

Policy information about studies involving animals; ARRIVE guidelines recommended for reporting animal research, and Sex and Gender in Research

Laboratory animals	6-8 week-old female mice (BALB/c background or the Nu/Nu nude mice) were used for all animal experiments described in this study. All mice used were purchased from the Vital River Laboratories. Mice were housed in groups of 5 mice per individually ventilated cage in a 12-h light–dark cycle (8:00–20:00 light; 20:00–8:00 dark), with constant room temperature (21 ± 1°C) and relative humidity (40-70%). All mice had access to food and water ad libitum.
Wild animals	The study did not involve wild animals.
Reporting on sex	Gender of mice has no preference for experimental results. Only female mice were used in this study. Sex information has not been collected. Considering that the disease model in this study was mainly a female disease, breast cancer, female animals were selected.

	In addition, this study involves a large number of immunological experiments, and individual differences in androgen levels will affect the detection indicators, so female animals were selected in this study.
Field-collected samples	The study did not involve samples collected from the field.
Ethics oversight	All mouse studies were conducted in accordance with the principles and procedures outlined in the Guide for the Care and Use of Laboratory Animals (Ministry of Health, China), and the protocol was approved by the Animal Experimental Ethical Inspection of Laboratory Animal Centre, Wenzhou Medical University.

Note that full information on the approval of the study protocol must also be provided in the manuscript.

Clinical data

Policy information about clinical studies

All manuscripts should comply with the ICMJEguidelines for publication of clinical research and a completedCONSORT checklist must be included with all submissions.

Clinical trial registration	*Provide the trial registration number from ClinicalTrials.gov or an equivalent agency.*
Study protocol	*Note where the full trial protocol can be accessed OR if not available, explain why.*
Data collection	*Describe the settings and locales of data collection, noting the time periods of recruitment and data collection.*
Outcomes	*Describe how you pre-defined primary and secondary outcome measures and how you assessed these measures.*

Dual use research of concern

Policy information about dual use research of concern

Hazards

Could the accidental, deliberate or reckless misuse of agents or technologies generated in the work, or the application of information presented in the manuscript, pose a threat to:

No	Yes	
☐	☐	Public health
☐	☐	National security
☐	☐	Crops and/or livestock
☐	☐	Ecosystems
☐	☐	Any other significant area

Experiments of concern

Does the work involve any of these experiments of concern:

No	Yes	
☐	☐	Demonstrate how to render a vaccine ineffective
☐	☐	Confer resistance to therapeutically useful antibiotics or antiviral agents
☐	☐	Enhance the virulence of a pathogen or render a nonpathogen virulent
☐	☐	Increase transmissibility of a pathogen
☐	☐	Alter the host range of a pathogen
☐	☐	Enable evasion of diagnostic/detection modalities
☐	☐	Enable the weaponization of a biological agent or toxin
☐	☐	Any other potentially harmful combination of experiments and agents

ChIP-seq

Data deposition

☐ Confirm that both raw and final processed data have been deposited in a public database such as GEO.

☐ Confirm that you have deposited or provided access to graph files (e.g. BED files) for the called peaks.

Data access links<br>*May remain private before publication.*	*For "Initial submission" or "Revised version" documents, provide reviewer access links. For your "Final submission" document, provide a link to the deposited data.*
Files in database submission	*Provide a list of all files available in the database submission.*

Genome browser session
(e.g. UCSC)

Provide a link to an anonymized genome browser session for "Initial submission" and "Revised version" documents only, to enable peer review. Write "no longer applicable" for "Final submission" documents.

Methodology

Replicates

Describe the experimental replicates, specifying number, type and replicate agreement.

Sequencing depth

Describe the sequencing depth for each experiment, providing the total number of reads, uniquely mapped reads, length of reads and whether they were paired- or single-end.

Antibodies

Describe the antibodies used for the ChIP-seq experiments; as applicable, provide supplier name, catalog number, clone name, and lot number.

Peak calling parameters

Specify the command line program and parameters used for read mapping and peak calling, including the ChIP, control and index files used.

Data quality

Describe the methods used to ensure data quality in full detail, including how many peaks are at FDR 5% and above 5-fold enrichment.

Software

Describe the software used to collect and analyze the ChIP-seq data. For custom code that has been deposited into a community repository, provide accession details.

Flow Cytometry

Plots

Confirm that:

- [x] The axis labels state the marker and fluorochrome used (e.g. CD4-FITC).
- [x] The axis scales are clearly visible. Include numbers along axes only for bottom left plot of group (a 'group' is an analysis of identical markers).
- [x] All plots are contour plots with outliers or pseudocolor plots.
- [x] A numerical value for number of cells or percentage (with statistics) is provided.

Methodology

Sample preparation

For FACS of tumour-infiltrating lymphocytes, tumours were collected on day 13. The tumors were dissected from the surrounding fascia, weighted, minced into pieces by a gentleMACS Dissociator (Miltenyi Biotec). Cell clumps were removed through a 100-μm cell strainer to obtain single-cell suspensions. The suspension was centrifuged, and the cell pellets were washed twice with cell staining buffer (Biolegend).

Instrument

Agilent Novocyte FACS

Software

FlowJo software package (version 10.4.0)

Cell population abundance

No sorting was performed.

Gating strategy

FSC/SSC gates were used to select mononuclear cells. FSC/FSH gates were then used to gate on single cells. Cells were then gated on live cells. Live cells were further gated on CD45+ leukocytes and then different populations of immune cells based on their expression of distinct markers. When needed, isotype control antibody staining was used to define positive/negative cell populations.

- [x] Tick this box to confirm that a figure exemplifying the gating strategy is provided in the Supplementary Information.

Magnetic resonance imaging

Experimental design

Design type

Indicate task or resting state; event-related or block design.

Design specifications

Specify the number of blocks, trials or experimental units per session and/or subject, and specify the length of each trial or block (if trials are blocked) and interval between trials.

Behavioral performance measures

State number and/or type of variables recorded (e.g. correct button press, response time) and what statistics were used to establish that the subjects were performing the task as expected (e.g. mean, range, and/or standard deviation across subjects).

Acquisition

Imaging type(s) | *Specify: functional, structural, diffusion, perfusion.*

Field strength | *Specify in Tesla*

Sequence & imaging parameters | *Specify the pulse sequence type (gradient echo, spin echo, etc.), imaging type (EPI, spiral, etc.), field of view, matrix size, slice thickness, orientation and TE/TR/flip angle.*

Area of acquisition | *State whether a whole brain scan was used OR define the area of acquisition, describing how the region was determined.*

Diffusion MRI ☐ Used ☐ Not used

Preprocessing

Preprocessing software | *Provide detail on software version and revision number and on specific parameters (model/functions, brain extraction, segmentation, smoothing kernel size, etc.).*

Normalization | *If data were normalized/standardized, describe the approach(es): specify linear or non-linear and define image types used for transformation OR indicate that data were not normalized and explain rationale for lack of normalization.*

Normalization template | *Describe the template used for normalization/transformation, specifying subject space or group standardized space (e.g. original Talairach, MNI305, ICBM152) OR indicate that the data were not normalized.*

Noise and artifact removal | *Describe your procedure(s) for artifact and structured noise removal, specifying motion parameters, tissue signals and physiological signals (heart rate, respiration).*

Volume censoring | *Define your software and/or method and criteria for volume censoring, and state the extent of such censoring.*

Statistical modeling & inference

Model type and settings | *Specify type (mass univariate, multivariate, RSA, predictive, etc.) and describe essential details of the model at the first and second levels (e.g. fixed, random or mixed effects; drift or auto-correlation).*

Effect(s) tested | *Define precise effect in terms of the task or stimulus conditions instead of psychological concepts and indicate whether ANOVA or factorial designs were used.*

Specify type of analysis: ☐ Whole brain ☐ ROI-based ☐ Both

Statistic type for inference
(See Eklund et al. 2016) | *Specify voxel-wise or cluster-wise and report all relevant parameters for cluster-wise methods.*

Correction | *Describe the type of correction and how it is obtained for multiple comparisons (e.g. FWE, FDR, permutation or Monte Carlo).*

Models & analysis

n/a	Involved in the study
☐	☐ Functional and/or effective connectivity
☐	☐ Graph analysis
☐	☐ Multivariate modeling or predictive analysis

Functional and/or effective connectivity | *Report the measures of dependence used and the model details (e.g. Pearson correlation, partial correlation, mutual information).*

Graph analysis | *Report the dependent variable and connectivity measure, specifying weighted graph or binarized graph, subject- or group-level, and the global and/or node summaries used (e.g. clustering coefficient, efficiency, etc.).*

Multivariate modeling and predictive analysis | *Specify independent variables, features extraction and dimension reduction, model, training and evaluation metrics.*
